# Does conservative kidney management offer a quantity or quality of life benefit compared to dialysis? A systematic review

**DOI:** 10.1186/s12882-021-02516-6

**Published:** 2021-09-11

**Authors:** Buur Louise Engelbrecht, Madsen Jens Kristian, Eidemak Inge, Krarup Elizabeth, Lauridsen Thomas Guldager, Taasti Lena Helbo, Finderup Jeanette

**Affiliations:** 1 Department of Renal Medicine, Aarhus University Hospital, Palle Juul-Jensens Boulevard 99, 8200 Aarhus, Denmark; 2 Department of Clinical Medicine, Aarhus University, Aarhus, Denmark; 3 Department of Public Health, Aarhus University, Aarhus, Denmark; 4 Department of Palliative Medicine, Rigshospitalet, Copenhagen, Denmark; 5 Department of Renal Medicine, Herlev and Gentofte Hospital, Herlev, Denmark; 6 Department of Renal Medicine, Aalborg University Hospital, Aalborg , Denmark; 7 Department of Medicine, North Zealand Hospital, Hillerød, Denmark

**Keywords:** Stage 5 chronic kidney disease, End-stage kidney disease, Dialysis, Conservative kidney management, Mortality, Quality of life

## Abstract

**Background:**

Patients with stage 5 chronic kidney disease (CKD5) collaborate with their clinicians when choosing their future treatment modality. Most elderly patients with CKD5 may only have two treatment options: dialysis or conservative kidney management (CKM). The objective of this systematic review was to investigate whether CKM offers a quantity or quality of life benefit compared to dialysis for some patients with CKD5.

**Methods:**

The databases MEDLINE, EMBASE, the Cochrane Library, and CINAHL were systematically searched for studies comparing patients with CKD5 who had chosen or were treated with either CKM or dialysis. The primary outcomes were mortality and quality of life (QoL). Hospitalization, symptom burden, and place of death were secondary outcomes. For studies reporting hazard ratios, pooled values were calculated, and forest plots conducted.

**Results:**

Twenty-five primary studies, all observational, were identified. All studies reported an increased mortality in patients treated with CKM (pooled hazard ratio 0.47, 95 % confidence interval 0.34–0.65). For patients aged ≥ 80 years and for elderly individuals with comorbidities, results were ambiguous. In most studies, CKM seemed advantageous for QoL and secondary outcomes. Findings were limited by the heterogeneity of studies and biased outcomes favouring dialysis.

**Conclusions:**

In general, patients with CKD5 who have chosen or are on CKM live for a shorter time than patients who have chosen or are on dialysis. In patients aged ≥ 80 years old, and in elderly individuals with comorbidities, the survival benefits of dialysis seem to be lost. Regarding QoL, symptom burden, hospitalization, and place of death, CKM may have advantages. Higher quality studies are needed to guide patients and clinicians in the decision-making process.

**Supplementary Information:**

The online version contains supplementary material available at 10.1186/s12882-021-02516-6.

## Introduction

Patients with CKD5 have high mortality rates [1]. Most patients are older than 65 years old, and less than 20 % are eligible for a kidney transplant [2].

Many of these older patients with CKD5 are not eligible for a kidney transplant because they are too frail. Therefore, when dialysis is needed, their treatment options are haemodialysis (HD) or peritoneal dialysis (PD), often in the form of assisted automated PD (AAPD). Dialysis can be burdensome for various reasons, including exhausting travel, complications related to the treatment, or the fact that it is so time-consuming. Thus, while dialysis may prolong patients’ lives, it may adversely affect their QoL [3]. For some patients, CKM could be a viable alternative to dialysis. CKM is a treatment strategy that gives patients all the same treatments as those on dialysis, omitting only the dialysis itself [4].

Patients and clinicians may find it challenging to discuss and decide on a future treatment modality based on both the best evidence and the individual patient’s preferences. Various aspects have to be considered, which may lead to a complex decision-making process. What is important depends on individual patients and may include survival, QoL, symptom burden, hospitalization, or place of death. Studies of this topic are sparse and heterogeneous, which presents challenges for clinical practice.

Usually, clinical guidelines inform clinical practice [5]. An international guideline from 2012 [6] approaches the structure and process of CKM as an alternative treatment pathway for patients with CKD5 who choose not to pursue kidney replacement therapy. The guideline reports paucity in many of the areas reviewed. However, MEDLINE was the only database searched in establishing this guideline. A European guideline from 2016 [7] addresses the question: *What is the benefit of dialysis in frail and older patients?* The guideline discusses many important factors related to treatment decision-making such as mortality, QoL, symptom burden, and hospitalization. For some of the issues, however, only a few studies were identified. A more recent UK guideline from 2018 [8] also addresses this question, but includes only mortality as an outcome. Given the lack of clinical guidelines, systematic reviews are beneficial in summarizing the evidence in studies around a specific topic. A number of systematic reviews of varying quality were published at around the same time as the European guideline [9–12]. To date, no randomized controlled trials (RCTs) have been published on this topic.

Recognizing the quality of the European guideline from 2016, the objective of this systematic review was to investigate whether CKM involves quantity or quality of life compared to dialysis for some patients with CKD5 in terms of the outcomes of mortality, QoL, hospitalization, symptom burden, and place of death.

## Materials and methods

 The systematic review has been conducted as recommended by the Cochrane Collaboration [13]. The process and results have been documented in accordance with the preferred reporting items for systematic reviews and meta-analyses (PRISMA) statement for reporting systematic reviews [14, 15]. The protocol for this review was prospectively registered with the Danish Health Authority, and no changes have been made. The review has been conducted by a working group involving doctors and nurses in nephrology as part of the preparation of a national clinical guideline. They have been supported with input from an interprofessional reference group consisting of representatives from the Danish Kidney Association and the Danish professional societies for nephrology, specialized palliative care, geriatrics, and general practitioners.

Eligibility Criteria.

### Participants

Based on the question *Does conservative kidney management offer a quantity or quality of life benefit compared to dialysis?* this review examined studies including adults aged 18 years old and above who had been diagnosed with CKD5. Studies that included adults with stage 1–4 chronic kidney disease (CKD1-4) or children were excluded.

### Interventions

Studies investigating CKM interventions or any intervention defined as a treatment strategy without dialysis for patients with CKD5 were considered, including patients who had chosen or were receiving CKM.

### Comparators

The review included studies comparing interventions for patients who had chosen or were treated with HD or PD.

### Outcomes

The primary outcomes were mortality and QoL. Secondary outcomes were hospitalization, symptom burden, and place of death, defined as whether the location of death was in accordance with a patient’s preference.

#### Types of Studies Included

This review considered all study designs relevant for answering the PICO (Patient, Intervention, Comparison, Outcome) question, including secondary literature such as systematic reviews, clinical guidelines, and grey literature.

#### Information Sources

According to the pre-specified protocol, a comprehensive literature search was conducted by the working group in collaboration with a literary search specialist from October 9, 2018 to May 13, 2019. This searched for guidelines in English, Danish, Norwegian, and Swedish published in electronic databases. The databases searched were Guidelines International Network (G-I-N), NICE (UK), Scottish Intercollegiate Guidelines Network (SIGN), HTA database, SBU (Sweden), Socialstyrelsen (Sweden), Helsedirektoratet (Norway), Kunnskapssenteret (Norway), MEDLINE, EMBASE, and CINAHL. The databases searched for secondary literature as well as primary literature were MEDLINE, EMBASE, the Cochrane Library, and CINAHL (Additional file 1).

#### Search Strategy

A three-phase search strategy was used to locate eligible studies. First, a search for clinical guidelines was conducted, followed by a search for secondary literature (Cochrane reviews, systematic reviews, and meta-analyses). Finally, primary literature was searched without adding any time limitation to our search. The full search protocols are given in the supplementary material (see Additional file 1). The following search terms were used to identify primary studies: exp Kidney Failure, Chronic/ ((end stage or chronic) adj3 (kidney diseas* or kidney failure* or renal disease* or renal failure*)).ab,kf,ti. AND Conservative Treatment/Palliative Care/ ((conservative or supportive) adj3 (treatment* or management or care)).ab,kf,ti. (nondialy* or non-dialy*).ab,kf,ti. ((without or refus*) adj2 dialys*) .ab,kf,ti. AND “Quality of Life”/ exp Mortality/Patient Readmission/ (“quality of life” or qol or mortality).ab,kf,ti. place of death.ab,kf,ti. (symptom* adj2 burden*).ab,kf,ti. Life Expectancy/ life expectancy.ab,kf,ti. The search terms ‘palliative care’ and ‘supportive’ were included, as these terms by some researchers have been used synonymously with CKM in a broader sense than end-of-life care.

#### Study Selection

 The process of selecting studies was administered through the systematic review management tool Covidence [16]. Titles were checked for duplicates when entering the eligible literature into Covidence. Two authors independently screened the titles and abstracts of the remaining studies for full-text retrieval. Similarly, two authors independently assessed full-text eligibility for inclusion. Discrepancies in judgement were resolved by consensus.

#### Data Extraction

All relevant data and outcomes (mortality, QoL, hospitalization, symptom burden, and place of death) for the PICO question were extracted from each study by two authors independently. Consensus was reached regarding any discrepancies.

#### Risk of Bias within Studies

To assess the quality of the studies selected, the Risk Of Bias In Non-randomized Studies of Interventions (ROBINS-I) tool was used because only observational studies were included. Assessment was carried out at outcome level and summarized.

#### Risk of Bias across Studies

The Grading of Recommendations Assessment, Development and Evaluation (GRADE) methodology was used to assess the quality of the body of evidence for each relevant outcome in the selected studies [17].

#### Data Synthesis

 The data extracted from the selected studies was entered into the Review Manager (RevMan5) software [18], used for preparing and updating Cochrane Reviews. Where comparable effect estimates and measures of variance (standard deviation, 95 % confidence intervals) were available, data were pooled using an inverse variance random effect model [19] to conduct a meta-analysis, presented as a forest plot. The overall heterogeneity of the studies was tested using the I^2^, indicating the variation across studies that is due to heterogeneity, where heterogeneity is indicated by I^2^ over 75 % [19]. The random effect model was chosen due to an expected heterogeneous effect [19]. Mostly, relevant findings are presented narratively. Means with standard deviations (SD), mean differences, and means with confidence intervals at a 95 % confidence level (95 % CI) were gathered for continuous data where possible. Hazard ratios (HR), relative risks (RR), and odds ratios (OR) with 95 % CI were collected from dichotomous data.

## Results

**Fig. 1:**
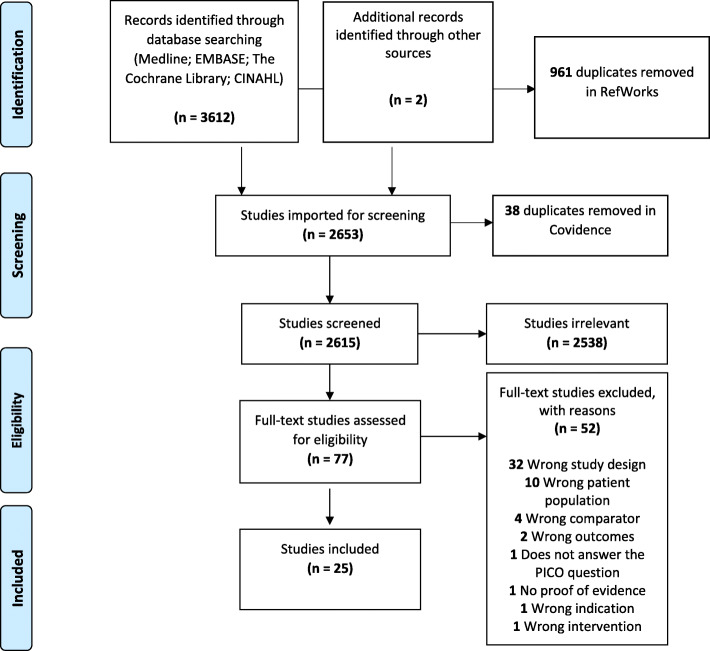
Flow chart for primary literature search

After removal of duplicates, screening of titles and abstracts, and subsequent full-text assessment, a total of one guideline, four systematic reviews, and 25 primary studies were identified. The flow diagram in Fig. 1 gives details of the primary literature search process. Flow diagrams for the guideline search and review search are included in the supplementary material (Additional file 2).

### Guideline and Systematic Reviews

The European guideline [7] covers the evidence relevant for our PICO question until the end of the guideline’s literature search in May 2016. All four of the systematic reviews identified [9, 10, 12, 20] synthesized the evidence. Not all the outcomes selected for the PICO question in this review were discussed in the earlier reviews. Two studies conducted meta-analyses, one including hospitalization and both including mortality as outcomes [9, 12]. In the supplementary material, we have provided an overview of the primary studies included in our review compared to studies included in the European guideline and the four systematic reviews selected (Additional file 3). The supplementary material also provides a list of studies present in the guideline or reviews that we did not include, with references and reasons for exclusion (Additional file 4).

### Primary Literature

Table 1 presents basic characteristics and data extraction for the 25 primary studies selected. All studies were observational: 11 prospective cohort studies [3, 21–30]; 10 retrospective cohort studies [31–40]; and 4 cross-sectional studies [41–44]. In general, patients on CKM were older and, when reported, had more comorbidities and poorer functional status compared to other groups. Nine of the included studies were conducted in UK [3, 22, 24, 25, 31, 39–41, 43], four in Hong Kong [28, 36, 38, 44] and three studies in Australia [27, 29, 43] as well as in the Netherlands [21, 32, 33]. The heterogeneity between studies in relation to interventions, comparators, statistical analyses, and treatment modalities affected the possibility of conducting meta-analyses for all outcomes except mortality and hospitalization. Even for these two outcomes, not all studies reported data suitable for meta-analyses. The quality assessments using ROBINS-I are presented in the supplementary material (Additional file 5), showing that only three studies had a low risk of bias, 12 studies had a moderate risk of bias, nine studies had a serious risk of bias, and one study had a critical risk of bias. The directness of bias for most studies was unpredictable or in favour of dialysis.

**Table 1:** Table with Details of the Primary Studies Selected for Inclusion

Author, year of publication, country	Population	Age (years)^1^	Intervention, n	Comparator, n	Main outcome measures and results^1^
**Almutary, 2016, Saudi Arabia **[42]	Non-dialysis group (CKD4 & CKM (CKD5)) Dialysis group (HD & PD)	Total group, 48.29 (± 14.86) CKM, (CKD5), 51.84 ± 15.11 HD, 47.71 ± 14.46 PD, 43.08 ± 15.09	CKM (CKD5), 38	HD, 287 PD, 42	*Symptom burden* (CKD-SBI) Dialysis vs. CKM (CKD5): HD: 23.36 ± 16.99; PD: 12.04 ± 6.58, vs. CKM (CKD5): 8.1 ± 8.04; *p* < 0.001
**Brown, 2015, Australia **[29]	CKD4 & CKD5	CKM, 82 ± 9 Pre-dialysis, 69 ± 14	CKM, 122	Pre-dialysis, 273 HD, 55 PD, 37	*Mortality* Dialysis vs. CKM, HR: 0.30 (95 % CI: 0.13–0.67); *p* = 0.003. Symptom burden & Quality of life No difference over time in groups.
**Carson, 2009, UK **[24]	CKD5	Age ≥ 70 at inclusion CKM, mean 81.6; median 83 Dialysis, mean 76.4; median 75	CKM, 29	Dialysis, 173	*Hospitalization rate* Hospital days/patient days survived: CKM, 4.3 ± 0.26; RRT, 6.9 ± 0.71 *Place of death* Home/hospice: CKM, 40 %; Dialysis 21 %, Hospital: CKM, 36 %; Dialysis 70 %
**Chandna**, **2011, UK **[40]	CKD5	CKM, 77.5 ± 7.6 Dialysis, 58.5 ± 15.0	CKM, 155	Dialysis, 689	*Mortality* Mortality in patients aged > 75: CKM vs. dialysis, HR: 1.18 (95 % CI: 0.79–1.76); *p* = 0.428. Months of survival in patients with comorbidity score > 4: Dialysis, 25.8 ± 4.4(SE); CKM, 20.4 ± 2.4(SE); *p* = 0.83.
**Da Silva-Gane, 2012, UK **[3]	CKD4-5	HD, 60.6 ± 14.9 PD, 48.0 ± 15.6 CKM, 77.5 ± 6.5	CKM, 30	HD,80 PD, 44	*Mortality* HD vs. CKM, HR 0.47 (95 % CI: 0.20–1.10); *p* = 0.08 PD vs. CKM, HR: 0.39 (95 % CI: 0.10–1.48); *p* = 0.17 *Quality of life* No difference over time in groups.
**Hussain**, **2013, UK **[39]	CKD5	Age > 70 at inclusion	CKM, 172	Dialysis, 269	*Hospitalization* Dialysis vs. CKM, RR 1.6 (95 % CI: 1.14–2.25), *p* < 0.05.
**Iyasere**, **2019, UK **[41]	CKD5	Median age (IQR) CKM, 83 (80–88) aAPD, 81 (79–88) HD, 82 (78–85)	CKM, 28	HD, 28 PD, 28	*Quality of life* SF-12, PCS: CKM, 28.9 ± 10.0; dialysis, 29.2 ± 8.3; *p* = 0.90 SF-12, MCS: CKM, 46.3 ± 12.1; dialysis, 49.9 ± 12.9; *p* = 0.28
**Joly**, **2003, France **[23]	CKD4-5	Age ≥ 80 at inclusion CKM, 84.1 ± 2.9 Dialysis, 83.2 ± 2.9	CKM, 43	Dialysis, 101	*Mortality* CKM, 8.9 months (95 % CI: 4–10); dialysis, 28.9 months (95 % CI: 24–38); *p* < 0.0001
**Kwok**, **2016, Hong Kong **[38]	CKD5	Age ≥ 65 at inclusion CKM, 79.6 ± 6.8 Dialysis, 74.2 ± 6.1	CKM, 432	Dialysis, 126	*Mortality* CKM, 10.0 months (95 % CI: 8.3–11.7); dialysis, 44.6 months (95 % CI: 37.3–51.9); *p* < 0.001.
**Murtagh**, **2007, UK **[31]	CKD5	Age > 75 at inclusion CKM, 81.36 Dialyse, 78,17	CKM, 77	Dialysis, 52	*Mortality* CKM vs. dialysis, HR: 2.90 (95 % CI: 1.60–5.26); In patients with comorbidity score > 2: Dialysis vs. CKM: log rank statistic < 0.001, df 1, p = 0.98, In patients with ischaemic heart disease: Dialysis vs. CKM: log rank statistic 1.46, df 1, *p* < 0.27.
**Raman**, **2018, UK **[22]	CKD5	Dialysis, 78.9 ± 2.8 CKM, 83.7 ± 4.2	CKM, 81	Dialysis, 123	*1-year survival* Dialysis vs. CKM, OR 0.38 (95 % CI: 0.09–1.60); *p* = 0.19 *Hospitalization* Number of admission days (median (IQR)): CKM, 0.8 (0.0-8.7); dialysis, 2.2 (0.7–14.7); *p* = 0.005.
**Reindl-Schwaighofer, 2017, Austria **[37]	CKD5	Age > 65 at inclusion HD, 74.06 ± 5.78 CKM, 81.22 ± 7.23	CKM, 174	HD, 8622	*Mortality* HD vs. CKM, HR: 0.23 (95 % CI: 0.18–0.29); *p* < 0.001.
**Seow, 2013, Singapore **[30]	CKD5	*Median age (IQR)*: CKM, 78 (40–70) Dialysis, 71 (50–80)	CKM, 63	Dialysis, 38	*Quality of life* No difference over time in groups.
**Shah**, **2019, Australia & UK **[43]	CKD5	Age ≥ 75 at inclusion *Median age (IQR)*: Dialysis, 81 (78–84) CKM, 83 (81–87) *Age ≤ 81, n (%)*: Dialysis, 50 (6) CKM, 19 (41) *Age > 81, n (%)*: Dialysis, 33 (40) CKM, 27 (59)	CKM, 46	Dialysis, 83	*Quality of life* Dialysis vs. CKM, adjusted differences in KDQOL-36 scores (95 % CI): KDQOL-burden of disease: -28.59 (-41.77 to -15.42); *p* < 0.001 KDQOL-symptoms of disease: -5.93 (-14.61 to 2.73); *p* = 0.18 KDQOL-effects of disease: -16.49 (-25.98 to -6.99); *p* < 0.001
**Shum**, **2014, Hong Kong **[36]	CKD5	Age 65–90 at inclusion Overall age, 73.8 ± 5.4 CKM, 75.3 ± 5.7 PD, 73.4 ± 5.3	CKM, 42	PD, 157	*Mortality* PD vs. CKM, HR: 0.46 (95 % CI 0.31–0.68), *p* < 0.001. *Hospitalization* Days per person year, median [IQR] PD, 16.17 [6.29–43.32] vs. CKM, 38.01 [6.75–76.56]; *p* = 0.03
**Smith**, **2003, UK **[25]	CKD5	CKM (palliative care population), 71 ± 12; Dialysis, 59 ± 15	CKM, 63	Dialysis, 258	*Mortality* Dialysis, median survival 8.3 months; CKM, median survival 6.3 months; N.S. *Place of death* Deaths at home or in a hospice: CKM, 22 of 34 deaths (65 %) Dialysis, 11 of 41 deaths (27 %); *p* = 0.001
**Tam-Tham**, **2018, Canada **[35]	CKD5	Age ≥ 65 at inclusion *Age 65 to < 75 (n, %)* Dialysis, 228 (45.6) CKM, 45 (13.3) *Age 75 to < 85 (n, %)* Dialysis, 220 (44.0) CKM, 143 (42.3) *Age ≥ 85 (n, %)* Dialysis, 52 (10.4) CKM, 150 (44.4)	CKM, 338	Dialysis, 500	*Mortality* Dialysis vs. CKM (0–3 years), HR: 0.56 (95 % CI: 0.44–0.71); *p* < 0.001 Dialysis vs. CKM (after 3 years), HR: 1.98 (95 % CI: 1.16–3.37); *p* = 0.12 *Hospitalization* Dialysis vs. CKM, HR: 1.40 (95 % CI: 1.16–1.69), *p* = 0.001
**Tan**, **2017, Australia **[27]	CKD5	Age > 65 at inclusion CKM, 84 Dialysis, 73	CKM, 8	Dialysis, 12	*Symptom burden* Change (improvement) in mean symptom POS-score over 6 months: CKM, 1.5; dialysis, 7.58; *p* < 0.002.
**Teo**, **2010, Singapore **[26]	CKD5	CKM, 67.4 ± 11.8 HD, 58.7 ± 12.9	CKM, 16	HD, 102	*Mortality* CKM, HR: 2.29 (95 % CI: 1.16–4.45); HD, HR: 0.59 (95 % CI: 0.33–1.05); *p* = 0.042
**Teruel**, **2015, Spain **[34]	CKD5	Median age (IQR) Dialysis, 68 (54,76) CKM, 83 (78,86)	CKM, 90	Dialysis, 142	*Mortality* CKM, 8.2/100 patient months; Dialysis, 0.6/100 patient months; *p* < 0.001
**van Loon**, **2019, the Netherlands **[21]	CKD5	Age ≥ 65 at inclusion CKM, 82 ± 6 Dialysis, 75 ± 7	CKM, 89	Dialysis, 192	*12-month survival* CKM vs. dialysis, HR: 2.12 (95 % CI: 1.12–4.03); *p* = 0.02 *In patients < 80 years old*, HR: 5.05 (95 % CI: 1.90–13.50); *p* < 0.01 *In patients ≥ 80 years old*, HR: 1.30 (95 % CI: 0.58–2.91); *p* = 0.53 *Six-month quality of life* EQ-5D Index, mean (SE) change within group: CKM, 0.047 (0.022); *p* < 0.01 Dialysis, 0.026 (0.014); *p* = 0.10 Between group difference, *p* < 0.01 *Hospitalization* Median number [IQR] of admissions: CKM, 1 [1-5]; Dialysis, [1-4]; *p* = 0.27 *Hospitalization* Median number [IQR] of admission days: Dialysis, 7 [3-15]; CKM, 4 [2-12]; *p* = 0.22
**Verberne**, **2018, the Netherlands **[33]	CKD5	CKM, 82.6 ± 4.5 Dialysis, 76.2 ± 4.4	CKM, 126	Dialysis, 240	*Mortality* CKM vs. dialysis, HR: 1.67 (95 % CI: 1.19–2.35), *p* = 0.003 Median [IQR] survival in years in patients ≥ 70 years old: CKM, 1.3 [0.5–2.5]; dialysis, 3.1 [1.7–6.4]; *p* < 0.001 Median [IQR] survival in years in patients ≥ 80 years old: CKM, 2.3 [1.3–3.7]; dialysis, 2.9 [1.9-6.0]; *p* = 0.13 *Quality of life* No difference between groups
**Verberne**, **2016, the Netherlands **[32]	CKD5	CKM, 83 ± 4.5 Dialysis, 76 ± 4.4	CKM, 107	Dialysis, 204	*Mortality* Dialysis vs. CKM, HR: 0.62 (95 % CI: 0.42–0.92), *p* = 0.02. Median [IQR] survival in years in patients ≥ 80 years old: CKM, 1.4 [0.7-3.0]; dialysis, 2.1 [1.5–3.4]; *p* = 0.08.
**Yong**, **2009, Hong Kong **[44]	CKD5	CKM, 73.1 ± 7.1 Dialysis, 58.2 ± 11.4	CKM, 45	Dialysis, 134	*Symptom burden* Number of symptoms CKM, 8.2 ± 3.9; dialysis 9.3 ± 4.7, *p* = 0.243
**Yuen**, **2016, Hong Kong **[28]	CKD5	CKM, 76.8 ± 9.1 Dialysis, 58.6 ± 12.6	CKM, 335	Dialysis, 265	*One-year survival (%)* CKM, 57.3 ± 2.9; dialysis, 89.7 ± 2.1 *3-year survival (%)* CKM, 16 ± 2.7; dialysis, 74.6 % ± 3.4

*CKD4 and CKD5* stage 4 and 5 chronic kidney disease; *CKM* conservative kidney management; *HD* haemodialysis; *PD* peritoneal dialysis; *AAPD* assisted automated peritoneal dialysis; *IQR* interquartile range; *HR* hazard ratio; *eGFR* estimated glomerular filtration rate; *SE* standard error; *RR* relative risk; *CKD-SBI* The CKD Symptom Burden Index; *SF-12* short form 12; *MCS* mental component summary; *PCS* physical component summary; *KDQOL* Kidney Disease Quality of Life; *POS* Palliative Outcome Scale; *EQ-5D* European Quality of life-5 Dimensions

^1^Unless otherwise noted, values are expressed as mean ± SD

#### Mortality

**Fig. 2:**
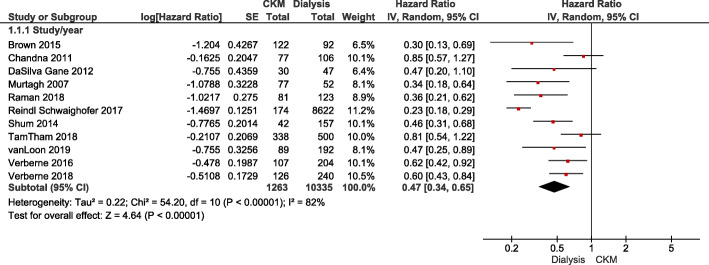
Forest plots of comparison: CKM versus dialysis for outcome: mortality

In total, 18 of both prospective and retrospective primary observational studies comparing CKM and dialysis, patients on CKM had higher mortality rates [3, 21–26, 28, 29, 31–40]. A meta-analysis is presented in Fig. 2. In a study of patients ≥ 75 years old, the higher mortality rate for those on CKM compared to dialysis was significantly reduced in patients with high comorbidities, especially ischemic heart disease [31]. For patients ≥ 80 years, the results seem conflicting. One study investigating octogenarians reported higher mortality for patients on CKM [23]. In contrast, three other studies found overall that mortality was equal for patients ≥ 80 years old [21, 32, 33].

#### Quality of life

One prospective cohort study found that patients with CKD5 on CKM had poorer self-reported QoL at baseline compared to patients on dialysis, but only with a borderline significance (p = 0.05) [21]. At a six-month follow-up, self-reported QoL was higher among patients on CKM compared to those on dialysis (p < 0.01). Another prospective cohort study found no difference in the mental health summary scores at baseline but significant difference in the physical health summary scores (p = 0.001). There was, however, no difference in QoL over time [3], a finding seen also in two other prospective cohort studies [29, 30] In a retrospective cohort study, there were no difference between patients managed conservatively and dialysis patients on physical and mental health summary scores [33]. The results of two cross-sectional studies were heterogeneous [41, 43]. One study found no difference in self-reported QoL (SF-36) between patients on CKM, HD, or PD [41]. Results from the other cross-sectional study varied depending on the tool used to measure QoL.

#### Symptom burden

In a small prospective study, commencement of dialysis in a younger cohort of elderly patients was associated with decrease in overall symptom burden [27]. In another prospective cohort study, however, no difference was found over time [29]. Two cross-sectional primary comparative studies found that patients on CKM had a lower symptom burden compared to patients on dialysis [42, 43]. A third cross-sectional study comparing a group of patients with CKD5 receiving CKM and patients on dialysis found overlapping symptom prevalence and intensity between the groups [44].

#### Hospitalization

**Fig. 3:**
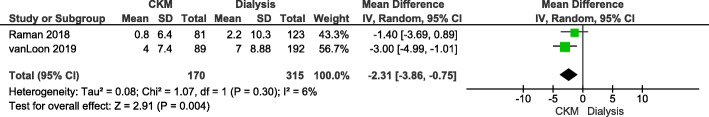
Forest plot of comparison: CKM versus dialysis for outcome: hospitalization – number of days spent in hospital

We identified three prospective [21, 22, 24] and three retrospective [35, 36, 39] primary cohort studies comparing the hospitalization of patients. Results were heterogeneous. Most studies found that patients on CKM had fewer hospital admissions or a significantly lower risk of hospitalization than patients on dialysis [22, 24, 35, 39]. Figure 3 shows a meta-analysis of the number of admission days. One study observed no difference in number of hospital admissions and number of days spent in hospital between groups [21]. Finally, one study comparing patients treated with CKM to patients on PD observed fewer days spent in hospital per person year for the patients treated with PD [36].

#### Place of death

Results from two primary studies indicate that patients on CKM more often die at home or in a hospice compared to patients on dialysis, who more often die in hospital [24, 25].

## Discussion

### Summary of Evidence

This review identified 25 studies comparing patients with CKD5 choosing or receiving CKM with those choosing or receiving dialysis to investigate the outcomes of mortality, QoL, symptom burden, hospitalization, and place of death. The studies were of variable quality, and there was substantial heterogeneity in presentation of the data, making it difficult to conduct an adequate meta-analysis for most outcomes.

Based on the available evidence, according to our review, CKM does not provide the same or extended survival in patients with CKD5 compared to dialysis. This is in line with previous systematic reviews [9, 12]. Overall, in contrast to CKM, dialysis is life prolonging. However, some studies indicate that the two treatment strategies may provide equal rates of mortality for patients who are 80 years old and above, or elderly patients with high comorbidities [21, 31, 32]. Thus, information on CKM may be considered in clinical practice in relation to this patient group. Some studies indicate that CKM may result in higher QoL compared to dialysis, and patients who receive CKM seem to have less hospitalization than patients on dialysis [21, 41–43]. Regarding symptom burden, results were conflicting. A recently published systematic review [45] concludes that in selected older patients, CKM has the potential to achieve similar QoL compared to a dialysis pathway [45].

Most of the studies identified compared patients on HD with those treated with PD or did not report details of dialysis modality. There was very limited data comparing patients on CKM with patients on PD. Our findings suggest that there may be differences between these two patient groups for the outcomes of symptom burden and hospitalization. Thus, these aspects should be considered in the decision-making process involved in choosing a patient’s preferred treatment strategy.

Only one study investigated whether preferred place of death for patients with CKD5 on either CKM or a dialysis pathway was congruent with their actual place of death [24]. The study indicated that patients on the CKM pathway more often die at home or in a hospice compared to patients on dialysis, who more often die in hospital. Studies of the general public and of patients with cancer have shown that most people would prefer to die at home [46, 47]. Based on our review, dying at home or in a hospice seems more likely to be the outcome for patients managed conservatively compared to patients on dialysis [24, 25]. Whether this result fulfils patients’ preferences was, however, unclear.

### Strengths and Limitations

 This review was conducted rigorously, using robust processes and relevant software tools. However, the study does have some limitations. All previous studies analysed were of observational design with variable sample size and quality and investigated patient groups that were heterogeneously defined. Furthermore, outcomes were assessed over different time periods. The quality of studies was reduced due to lead time bias when estimating mortality, and by confounders mostly favouring dialysis. Data heterogeneity restricted the use of meta-analysis. A high heterogeneity (I^2^ = 82 %) was found in the meta-analysis of mortality (See Fig. 2). One reason for this heterogeneity could be variation between countries. The total of included studies represents only 10 countries with more than a third of the studies having been conducted in UK. A sub-analysis of four studies from UK [3, 25, 31, 40] had a moderate heterogeneity (I^2^ = 68 %) with a pooled hazard ratio of mortality of 0.49; 95 % confidence interval 0.29–0.74. A sub-analysis of three studies from the Netherlands [21, 32, 33] had a low heterogeneity (I^2^ = 0 %) with a pooled hazard ratio of 0.59; 95 % confidence interval 0.46–0.74. A study determining the practice patterns of CKM in UK showed that CKM is acknowledged in all renal units. However, considerable variation was seen in how units described and delivered CKM [48]. We identified such variation also among the studies included in this review which may have caused selection bias and may have in some studies influenced the results in predictable way.

### Implications for Clinical Practice and Further Research

Discussion of treatment options with clinicians is crucial for patients with CKD5 regardless of their preferred modality of treatment. In a recent qualitative study from the UK, 20 patients receiving CKM were interviewed [49]. The patients’ experience was that clinicians avoided talking about diagnosis and prognosis related to their disease. The patients expressed a desire to receive information related to their disease and possible treatment choices. At the same time, however, they were ambivalent about receiving detailed knowledge on the progression of their disease. Although the evidence in our review relies on observational data, the results suggest a CKM pathway can be an acceptable alternative to dialysis for patients aged 80 years and above or elderly patients with comorbidities. Consequently, discussion of CKM as a future treatment modality with this group of patients is important. A Canadian survey from 2010 showed that 60 % of the patients receiving dialysis regretted having started the treatment [50]. The findings of one qualitative review indicated that patients with CKD were capable of prioritizing QoL and freedom over survival [51]. Based on the findings of this review, aspects of QoL, symptom burden, and hospitalization should be considered in the decision-making process when choosing the preferred treatment strategy.

To date, the evidence of outcomes for patients with CKD5 receiving dialysis compared to patients on a CKM pathway has been drawn from observational studies of varying quality, many of which were retrospective. No randomized controlled studies have yet been published in this area. For ethical reasons, conducting such studies may be very problematic or even impossible. Thus, future research may also have to rely mainly on observational studies. Such studies should be carefully planned, with a prospective design and a strict methodology to minimize bias and the influence of confounders. Comparative studies of patients on CKM or PD may provide a more nuanced basis for discussions of future treatment choices for patients with CKD5.

## Conclusions

 In this systematic review, we explored studies evaluating CKM as an alternative to dialysis for adult patients with CKD5 in relation to mortality, QoL, hospitalization, symptom burden, and place of death. Overall, patients with CKD5 on CKM have poorer survival compared to patients on dialysis. However, we observed that for patients aged 80 years and above, or elderly patients with severe comorbidities, the improved survival on dialysis over CKM appears to vanish. Despite some inconsistencies, the results suggest CKM has advantages compared to dialysis for the outcomes of QoL, hospitalization, symptom burden, and place of death. These findings should be addressed when discussing future treatment options with patients. More rigorously conducted studies are needed to establish a better base for such a decision-making process.

## Supplementary Information

**Additional file 1:**

**Additional file 2:**

**Additional file 3:**

**Additional file 4:**

**Additional file 5:**

## Data Availability

Data sharing is not applicable to this article as no dataset were generated or analysed during the current study.

## Abbreviations

CKD5: Stage 5 chronic kidney disease
CKM: Conservative kidney management
QoL: Quality of life
HD: Haemodialysis
PD: Peritoneal dialysis
AAPD: Assisted automated peritoneal dialysis
RCTs: Randomized controlled trials
PRISMA: Preferred reporting items for systematic reviews and meta-analyses
CKD 1–4: Stage 1–4 chronic kidney disease
PICO: Patient, Intervention, Comparison, Outcome
ROBINS-I: Risk Of Bias In Non-randomized Studies of Interventions tool
GRADE: The Grading of Recommendations Assessment, Development and Evaluation methodology
RevMan5: Review Manager software
SD: Standard deviations
95 % CI: 95 % confidence Interval
HR: Hazard ratios
RR: Relative risks
OR: Odds ratios (OR)
