# Correction: Does conservative kidney management offer a quantity or quality of life benefit compared to dialysis? a systematic review

**DOI:** 10.1186/s12882-022-02963-9

**Published:** 2022-11-02

**Authors:** Louise Engelbrecht Buur, Jens Kristian Madsen, Inge Eidemak, Elizabeth Krarup, Thomas Guldager Lauridsen, Lena Helbo Taasti, Jeanette Finderup

**Affiliations:** 1 Department of Renal Medicine, Aarhus University Hospital, Palle Juul-Jensens Boulevard 99, 8200 Aarhus, Denmark; 2 Department of Clinical Medicine, Aarhus University, Aarhus, Denmark; 3 Department of Public Health, Aarhus University, Aarhus, Denmark; 4 Department of Palliative Medicine, Rigshospitalet, Copenhagen, Denmark; 5 Department of Renal Medicine, Herlev and Gentofte Hospital, Herlev, Denmark; 6 Department of Renal Medicine, Aalborg University Hospital, Aalborg, Denmark; 7 Department of Medicine, North Zealand Hospital, Hillerød, Denmark

**Correction: BMC Nephrology (2021) 22:307**

10.1186/s12882-021-02516-6.

Following publication of the original article [1], we have been informed that first name and last name have been swapped for all the authors.

The author group has been updated above and the original article [1] has been corrected.
