# Supplementary material for: Does conservative kidney management offer a quantity or quality of life benefit compared to dialysis? A systematic review
Source: BMC Nephrol. 2021 Sep 11;22:307. doi: 10.1186/s12882-021-02516-6 (PMC8434727; doi:10.1186/s12882-021-02516-6)
Supplement: Supplementary file 1 — Additional file 1: [file 12882_2021_2516_MOESM1_ESM.docx]

## Additional file 1: Search Protocols for Guidelines, Primary and Secondary Literature

### Search Protocol – Guidelines

| **Project title/aspect** | Conservative kidney management in patients with stage 5 CKD PICO search on guidelines |
| --- | --- |
| **Last updated** | 12.10.2018 |

| **Background** | **PICO question** Should the adult patient with stage 5 CKD be offered a treatment strategy without dialysis (CKM) as an alternative to dialysis? |
| --- | --- |
| **Terms for search** | English: chronic kidney failure, chronic kidney disease, end stage renal disease, end stage renal failure, renal replacement therapy, chronic renal failure, chronic renal disease  Swedish: njursvikt, njursjukdomar  Norwegian: nyresvikt |
| **Inclusion and exclusion criteria** | Language: English, Danish, Norwegian, and Swedish  Year: 2008-2018 in the MEDLINE, EMBASE, and CINAHL databases  Population: Adults in the MEDLINE, EMBASE, and CINAHL databases  Publication types: Guidelines, practice guidelines |

**Search strategy PICO (guidelines)**

| Database | Interface | Date of search |
| --- | --- | --- |
| **G-I-N International** | https://www.g-i-n.net/ | 09.10.2018 |
| **NICE, UK** | https://www.nice.org.uk/ | 09.10.2018 |
| **Scottish Intercollegiate Guidelines Network (SIGN)** | http://www.sign.ac.uk/our-guidelines.html | 09.10.2018 |
| **HTA (CRD)** | http://www.crd.york.ac.uk/CRDWeb/ | 09.10.2018 |
| **SBU, Sweden** | http://www.sbu.se/sv/ | 09.10.2018 |
| **Socialstyrelsen, Sweden** | http://www.socialstyrelsen.se/riktlinjer/nationellariktlinjer | 09.10.2018 |
| **Helsedirektoratet, Norway** | https://helsedirektoratet.no/retningslinjer | 09.10.2018 |
| **Kunnskapssenteret, Norway** | https://www.fhi.no/oversikter/alle/ | 09.10.2018 |

| **MEDLINE** | Ovid MEDLINE(R) Epub ahead of print, in process and other non-indexed citations, Ovid MEDLINE(R) daily, and Ovid MEDLINE(R) <1946 to present> | 11.10.2018 |
| --- | --- | --- |
| **EMBASE** | Ovid EMBASE 1974 to 11.10.2018 | 11.10.2018 |
| **CINAHL** | EBSCO CINAHL with full text | 11.10.2018 |

**Search strategy**

**G-I-N International**

| Keywords | Hits | Comments |
| --- | --- | --- |
| Search for guidelines  (chronic kidney failure or chronic kidney disease or end stage renal disease or end stage renal failure or renal replacement therapy or chronic renal failure or chronic renal disease) | 41 (10) | Excluded: 17 obviously irrelevant 9 wrong language 5 NICE guidelines |

**NICE (UK)**

| Keywords | Hits | Comments |
| --- | --- | --- |
| Search for guidelines: clinical guidelines and NICE guidelines  (chronic kidney failure or chronic kidney disease or end stage renal disease or end stage renal failure or renal replacement therapy or chronic renal failure or chronic renal disease) | 7 (5) | Excluded:  2 obviously irrelevant |

**Scottish Intercollegiate Guidelines Network (SIGN)**

No relevant guidelines were found in this database.

**HTA Database (CRD database)**

| Keywords | Hits | Comments |
| --- | --- | --- |
| IN HTA  (chronic kidney failure or chronic kidney disease or end stage renal disease or end stage renal failure or renal replacement therapy or chronic renal failure or chronic renal disease) | 92 |  |

**SBU, Sweden**

| Keywords | Hits | Comments |
| --- | --- | --- |
| Search for terms:  Utvärderar  njursvikt or njursjukdomar | 1 (0) | Excluded:  1 obviously irrelevant |

**Socialstyrelsen, Sweden**

No relevant guidelines were found in this database.

**Helsedirektoratet, Norway**

No relevant guidelines were found in this database.

**Kunnskapssenteret, Norway**

| Keywords | Hits | Comments |
| --- | --- | --- |
| Nyresvikt | 8 (1) | Excluded:  7 obviously irrelevant |

**MEDLINE**

| 1 | exp Renal Insufficiency, Chronic/ | 104550 |
| --- | --- | --- |
| 2 | exp Renal Replacement Therapy/ | 192719 |
| 3 | ((chronic or “end stage”) adj2 (“kidney failure” or “renal failure” or “kidney disease” or “renal disease”)).ab,kf,ti. | 95466 |
| 4 | renal replacement therapy.ab,kf,ti. | 10787 |
| 5 | or/1-4 | 279442 |
| 6 | exp age groups/ not exp Adult/ | 1764516 |
| 7 | 5 not 6 | 265250 |
| 8 | limit 7 to (guideline or practice guideline) | 648 |
| 9 | limit 8 to (yr=“2008 -Current” and (danish or english or norwegian or swedish)) | 249 |

**EMBASE**

| 1 | exp chronic kidney failure/ | 75018 |
| --- | --- | --- |
| 2 | end stage renal disease/ | 2247 |
| 3 | exp renal replacement therapy/ | 170196 |
| 4 | ((chronic or “end stage”) adj2 (“kidney failure” or “renal failure” or “kidney disease” or “renal disease”)).ab,kw,ti. | 139833 |
| 5 | “renal replacement therapy”.ab,kw,ti. | 17632 |
| 6 | or/1-5 | 295164 |
| 7 | exp groups by age/ not exp adult/ | 2300406 |
| 8 | 6 not 7 | 278289 |
| 9 | (Guideline* or practice guideline* or clinical guideline*).ti,kw,pt. | 105639 |
| 10 | 8 and 9 | 2077 |
| 11 | limit 10 to ((danish or english or norwegian or swedish) and yr=“2008 -Current”) | 1247 |
| 12 | limit 11 to (conference abstract or conference paper or “conference review” or editorial or letter) | 276 |
| 13 | 11 not 12 | 971 |

**CINAHL**

| Search ID# | Search terms | Search options | Results |
| --- | --- | --- | --- |
| S9 | S8 | Limiters - Published Date: 20080101-30001231; Language: Danish, English, Norwegian, Swedish  Search modes - Boolean/Phrase | 63 |
| S8 | S7 | Limiters - Publication Type: Practice Guidelines  Search modes - Boolean/Phrase | 138 |
| S7 | S5 NOT S6 | Search modes - Boolean/Phrase | 27862 |
| S6 | (MH “Named Groups by Age+”) NOT (MH “Adult+”) | Search modes - Boolean/Phrase | 338596 |
| S5 | S1 OR S2 OR S3 OR S4 | Search modes - Boolean/Phrase | 29056 |
| S4 | renal replacement therapy | Search modes - Boolean/Phrase | 1983 |
| S3 | ((chronic or “end stage”) N2 (“kidney failure” or “renal failure” or “kidney disease” or “renal disease”)) | Search modes - Boolean/Phrase | 18012 |
| S2 | (MH “Renal Replacement Therapy+”) | Search modes - Boolean/Phrase | 15773 |
| S1 | (MH “Renal Insufficiency, Chronic+”) | Search modes - Boolean/Phrase | 13425 |

### Search Protocol – Primary Literature

| **Project title/aspect** | Conservative kidney management in patients with stage 5 CKD PICO search on primary literature |
| --- | --- |
| **Last updated** | 13.05.2019 |

| **Background** | **PICO question** Should the adult patient with stage 5 CKD be offered a treatment strategy without dialysis (CKM) as an alternative to dialysis? |
| --- | --- |
| **Inclusion and exclusion criteria** | Language: English, Danish, Norwegian, and Swedish  Year: See the search strategy for the PICO question below  Publication types: See the search strategy for the PICO question below (In the EMBASE searches, conference publications, editorials, and letters are excluded) |

**Search strategy**

**PICO**

| Database | Interface | Hits | Date of search |
| --- | --- | --- | --- |
| **MEDLINE** | Ovid MEDLINE(R) Epub ahead of print, in process, and other non-indexed citations, Ovid MEDLINE(R) daily, and Ovid MEDLINE(R) <1946 to 10.05.2019 | 828 | 13.05.2019 |
| **EMBASE** | Ovid EMBASE 1974 to 10.05.2019 | 1588 | 13.05.2019 |
| **Cochrane library** | http://cochranelibrary-wiley.com | 802 | 13.05.2019 |
| **CINAHL** | EBSCO CINAHL with full text | 391 | 13.05.2019 |

**MEDLINE**

| 1 | exp Kidney Failure, Chronic/ | 90030 |
| --- | --- | --- |
| 2 | ((end stage or chronic) adj3 (kidney diseas* or kidney failure* or renal disease* or renal failure*)).ab,kf,ti. | 101848 |
| 3 | 1 or 2 | 149846 |
| 4 | Conservative Treatment/ | 1795 |
| 5 | Palliative Care/ | 50630 |
| 6 | ((conservative or supportive) adj3 (treatment* or management or care)).ab,kf,ti. | 69646 |
| 7 | (nondialy* or non-dialy*).ab,kf,ti. | 3715 |
| 8 | ((without or refus*) adj2 dialys*).ab,kf,ti. | 1157 |
| 9 | or/4-8 | 124148 |
| 10 | “Quality of Life”/ | 175618 |
| 11 | exp Mortality/ | 358468 |
| 12 | Patient Readmission/ | 14701 |
| 13 | (“quality of life” or qol or mortality).ab,kf,ti. | 927738 |
| 14 | place of death.ab,kf,ti. | 1124 |
| 15 | (symptom* adj2 burden*).ab,kf,ti. | 3496 |
| 16 | Life Expectancy/ | 16683 |
| 17 | life expectancy.ab,kf,ti. | 28666 |
| 18 | ((multidisciplin* or multicomponent* or interdisciplin* or patient centered or person centered) adj3 (team* or care or program* or setting*)).ab,kf,ti. | 40122 |
| 19 | Patient Care Team/ | 62542 |
| 20 | or/10-19 | 1318212 |
| 21 | 3 and 9 and 20 | 1164 |
| 22 | limit 21 to (editorial or letter or meta-analysis or guideline or practice guideline or “review” or “systematic review”) | 290 |
| 23 | 21 not 22 | 874 |
| 24 | limit 23 to (danish or english or norwegian or swedish) | 828 |

**EMBASE**

| 1 | exp chronic kidney failure/ | 81611 |
| --- | --- | --- |
| 2 | ((end stage or chronic) adj3 (kidney diseas* or kidney failure* or renal disease* or renal failure*)).ab,kw,ti. | 150042 |
| 3 | 1 or 2 | 183524 |
| 4 | exp conservative treatment/ | 533116 |
| 5 | ((conservative or supportive) adj3 (treatment* or management or care)).ab,kw,ti. | 103567 |
| 6 | (nondialy* or non-dialy*).ab,kw,ti. | 4506 |
| 7 | ((without or refus*) adj2 dialys*).ab,kw,ti. | 1632 |
| 8 | or/4-7 | 605410 |
| 9 | exp “quality of life”/ | 460136 |
| 10 | exp mortality/ | 979007 |
| 11 | hospital readmission/ | 52466 |
| 12 | (“quality of life” or qol or mortality).ab,kw,ti. | 1376805 |
| 13 | place of death.ab,kw,ti. | 1721 |
| 14 | (symptom* adj2 burden*).ab,kw,ti. | 6775 |
| 15 | life expectancy/ | 45278 |
| 16 | life expectancy.ab,kw,ti. | 40424 |
| 17 | ((multidisciplin* or multicomponent* or interdisciplin* or patient centered or person centered) adj3 (team* or care or program* or setting*)).ab,kw,ti. | 66010 |
| 18 | or/9-17 | 1942275 |
| 19 | 3 and 8 and 18 | 3573 |
| 20 | limit 19 to (conference abstract or conference paper or “conference review” or editorial or letter or “review”) | 1864 |
| 21 | 19 not 20 | 1709 |
| 22 | limit 21 to (danish or english or norwegian or swedish) | 1588 |

**Cochrane Library**

| #1 | MeSH descriptor: [Kidney Failure, Chronic] explode all trees | 4343 |
| --- | --- | --- |
| #2 | (((end stage or chronic) NEAR/3 (kidney diseas* or kidney failure* or renal disease* or renal failure*))):ti,ab,kw (Word variations have been searched) | 66622 |
| #3 | #1 OR #2 | 66622 |
| #4 | MeSH descriptor: [Conservative Treatment] this term only | 78 |
| #5 | MeSH descriptor: [Palliative Care] this term only | 1498 |
| #6 | ((conservative or supportive) NEAR/3 (treatment* or management or care)):ti,ab,kw (Word variations have been searched) | 16128 |
| #7 | (nondialy* or non-dialy*):ti,ab,kw (Word variations have been searched) | 409 |
| #8 | ((without or refus*) NEAR/2 dialys*):ti,ab,kw (Word variations have been searched) | 131 |
| #9 | #4 OR #5 OR #6 OR #7 OR #8 | 18028 |
| #10 | MeSH descriptor: [Quality of Life] this term only | 21390 |
| #11 | MeSH descriptor: [Mortality] explode all trees | 12638 |
| #12 | MeSH descriptor: [Patient Readmission] this term only | 934 |
| #13 | (“quality of life” or qol or mortality):ti,ab,kw (Word variations have been searched) | 170553 |
| #14 | (place of death):ti,ab,kw (Word variations have been searched) | 1749 |
| #15 | (symptom* NEAR/2 burden*):ti,ab,kw (Word variations have been searched) | 947 |
| #16 | MeSH descriptor: [Life Expectancy] this term only | 121 |
| #17 | (life expectancy):ti,ab,kw (Word variations have been searched) | 12033 |
| #18 | ((multidisciplin* or multicomponent* or interdisciplin* or patient centered or person centered) NEAR/3 (team* or care or program* or setting*)):ti,ab,kw (Word variations have been searched) | 50261 |
| #19 | MeSH descriptor: [Patient Care Team] this term only | 1587 |
| #20 | #10 OR #11 OR #12 OR #13 OR #14 OR #15 OR #16 OR #17 OR #18 OR #19 | 215238 |
| #21 | #3 AND #9 AND #20 | 852 |
| - | CENTRAL | 802 |

**CINAHL**

| # | Query | Limiters/Expanders | Results |
| --- | --- | --- | --- |
| S21 | S20 | Limiters - Language: Danish, English, Norwegian, Swedish  Search modes - Boolean/Phrase | 391 |
| S20 | S3 AND S8 AND S19 | Search modes - Boolean/Phrase | 398 |
| S19 | S9 OR S10 OR S11 OR S12 OR S13 OR S14 OR S15 OR S16 OR S17 OR S18 | Search modes - Boolean/Phrase | 471941 |
| S18 | (MH “Multidisciplinary Care Team”) | Search modes - Boolean/Phrase | 38147 |
| S17 | (multidisciplin* or multicomponent* or interdisciplin* or patient centered or person centered) N3 (team* or care or program* or setting*) | Search modes - Boolean/Phrase | 74947 |
| S16 | life expectancy | Search modes - Boolean/Phrase | 11773 |
| S15 | (MH “Life Expectancy”) | Search modes - Boolean/Phrase | 6711 |
| S14 | symptom* N2 burden* | Search modes - Boolean/Phrase | 2252 |
| S13 | place of death | Search modes - Boolean/Phrase | 621 |
| S12 | “quality of life” or qol or mortality | Search modes - Boolean/Phrase | 380169 |
| S11 | (MH “Readmission”) | Search modes - Boolean/Phrase | 10990 |
| S10 | (MH “Mortality+”) | Search modes - Boolean/Phrase | 60355 |
| S9 | (MH “Quality of Life+”) | Search modes - Boolean/Phrase | 100671 |
| S8 | S4 OR S5 OR S6 OR S7 | Search modes - Boolean/Phrase | 47584 |
| S7 | (without or refus*) N2 dialys* | Search modes - Boolean/Phrase | 187 |
| S6 | nondialy* or non-dialy* | Search modes - Boolean/Phrase | 527 |
| S5 | (conservative or supportive) N3 (treatment* or management or care) | Search modes - Boolean/Phrase | 16528 |
| S4 | (MH “Palliative Care”) | Search modes - Boolean/Phrase | 31135 |
| S3 | S1 OR S2 | Search modes - Boolean/Phrase | 32175 |
| S2 | (end stage or chronic) N3 (kidney diseas* or kidney failure* or renal disease* or renal failure*) | Search modes - Boolean/Phrase | 32172 |
| S1 | (MH “Kidney Failure, Chronic+”) | Search modes - Boolean/Phrase | 18972 |

### Search Protocol – Secondary Literature

| **Project title/aspect** | Conservative kidney management in patients with stage 5 CKD PICO search on systematic reviews |
| --- | --- |
| **Last updated** | 20.12.2018 |

| **Background** | **PICO question** Should the adult patient with stage 5 CKD be offered a treatment strategy without dialysis (CKM) as an alternative to dialysis? |
| --- | --- |
| **Inclusion and exclusion criteria** | Language: English, Danish, Norwegian, and Swedish  Year: No limitations  Publication types: Systematic reviews and meta-analyses |

**Search strategy**

**PICO (secondary literature)**

| Database | Interface | Hits | Date of search |
| --- | --- | --- | --- |
| **MEDLINE** | Ovid MEDLINE(R) Epub ahead of print, in process, and other non-indexed citations, Ovid MEDLINE(R) daily, and Ovid MEDLINE(R) <1946 to 19.11.2018> | 218 | 20.11.2018 |
| **EMBASE** | Ovid EMBASE 1974 to 19.11.2018 | 555 | 20.11.2018 |
| **Cochrane Library** | https://www.cochranelibrary.com/advanced-search | 49 | 20.11.2018 |
| **CINAHL** | EBSCO CINAHL with full text | 57 | 20.11.2018 |

**MEDLINE**

| 1 | exp Kidney Failure, Chronic/ | 88885 |
| --- | --- | --- |
| 2 | exp Renal Replacement Therapy/ | 193366 |
| 3 | ((end stage or chronic) adj3 (kidney diseas* or kidney failure* or renal disease* or renal failure*)).ab,kf,ti. | 98280 |
| 4 | ((renal or kidney) adj3 replace*).ab,kf,ti. | 12752 |
| 5 | (hemodiafilt* or haemodiafilt* or hemodialys* or haemodialys*).ab,kf,ti. | 73417 |
| 6 | “dialys*”.ab,kf,ti. | 108337 |
| 7 | or/1-6 | 323451 |
| 8 | Conservative Treatment/ | 1382 |
| 9 | Palliative Care/ | 49567 |
| 10 | exp “Delivery of Health Care, Integrated”/ | 11414 |
| 11 | ((conservative or supportive or palliative) adj3 (treatment* or management or care)).ab,kf,ti. | 101448 |
| 12 | integrated care.ab,kf,ti. | 3829 |
| 13 | (nondialys* or non-dialys*).ab,kf,ti. | 2006 |
| 14 | or/8-13 | 146787 |
| 15 | exp “Outcome and Process Assessment (Health Care)”/ | 994646 |
| 16 | exp Prognosis/ | 1464148 |
| 17 | “Quality of Life”/ | 168693 |
| 18 | exp Mortality/ | 350195 |
| 19 | Patient Readmission/ | 13857 |
| 20 | exp Emotions/ | 218825 |
| 21 | Depression/ | 104868 |
| 22 | Clinical Decision-Making/ | 4424 |
| 23 | exp Decision Making/ | 181634 |
| 24 | (“quality of life” or qol or mortality or anxiety or depression).ab,kf,ti. | 1256315 |
| 25 | (decision* adj2 (regret* or conflict*)).ab,kf,ti. | 1456 |
| 26 | place of death.ab,kf,ti. | 1064 |
| 27 | (symptom* adj2 burden*).ab,kf,ti. | 3205 |
| 28 | Life Expectancy/ | 16358 |
| 29 | life expectancy.ab,kf,ti. | 27743 |
| 30 | or/15-29 | 3079261 |
| 31 | 7 and 14 and 30 | 1949 |
| 32 | (((systematic or method*) adj3 (review* or overview* or study or studies or search* or approach*)) or meta analy* or meta-analy* or metaanaly*).ti,ab,kw,sh,pt. | 701082 |
| 33 | (pooled adj1 (data or analys*)).ti,ab. | 15351 |
| 34 | 32 or 33 | 708804 |
| 35 | limit 31 to (meta-analysis or systematic reviews) | 132 |
| 36 | 31 and 34 | 183 |
| 37 | 35 or 36 | 235 |
| 38 | limit 37 to (danish or english or norwegian or swedish) | 218 |

**EMBASE**

| 1 | exp chronic kidney failure/ | 76577 |
| --- | --- | --- |
| 2 | exp renal replacement therapy/ | 171586 |
| 3 | ((end stage or chronic) adj3 (kidney diseas* or kidney failure* or renal disease* or renal failure*)).ab,kw,ti. | 144288 |
| 4 | ((renal or kidney) adj3 replace*).ab,kw,ti. | 20660 |
| 5 | (hemodiafilt* or haemodiafilt* or hemodialys* or haemodialys*).ab,kw,ti. | 101218 |
| 6 | “dialys*”.ab,kw,ti. | 146293 |
| 7 | or/1-6 | 357950 |
| 8 | exp conservative treatment/ | 518823 |
| 9 | integrated health care system/ | 10109 |
| 10 | ((conservative or supportive or palliative) adj3 (treatment* or management or care)).ab,kw,ti. | 151446 |
| 11 | integrated care.ab,kw,ti. | 5018 |
| 12 | (nondialys* or non-dialys*).ab,kw,ti. | 2786 |
| 13 | or/8-12 | 610360 |
| 14 | outcome assessment/ | 450724 |
| 15 | health care quality/ | 226697 |
| 16 | exp prognosis/ | 633482 |
| 17 | exp “quality of life”/ | 440312 |
| 18 | exp mortality/ | 945831 |
| 19 | hospital readmission/ | 48623 |
| 20 | exp emotion/ | 509367 |
| 21 | exp depression/ | 419476 |
| 22 | clinical decision making/ | 37371 |
| 23 | exp decision making/ | 328932 |
| 24 | life expectancy/ | 43803 |
| 25 | (“quality of life” or qol or mortality or anxiety or depression).ab,kw,ti. | 1803105 |
| 26 | (decision* adj2 (regret* or conflict*)).ab,kw,ti. | 2085 |
| 27 | place of death.ab,kw,ti. | 1643 |
| 28 | (symptom* adj2 burden*).ab,kw,ti. | 6168 |
| 29 | life expectancy.ab,kw,ti. | 38799 |
| 30 | or/14-29 | 3908294 |
| 31 | 7 and 13 and 30 | 6606 |
| 32 | (((systematic or method*) adj3 (review* or overview* or study or studies or search* or approach*)) or meta analy* or meta-analy* or metaanaly*).ti,ab,kw,sh,pt. | 1232802 |
| 33 | (pooled adj1 (data or analys*)).ti,ab. | 23568 |
| 34 | 32 or 33 | 1245297 |
| 35 | 31 and 34 | 740 |
| 36 | limit 31 to (meta-analysis or “systematic review”) | 277 |
| 37 | 35 or 36 | 770 |
| 38 | limit 37 to (danish or english or norwegian or swedish) | 751 |
| 39 | limit 38 to (conference abstract or conference paper or “conference review” or editorial or letter) | 196 |
| 40 | 38 not 39 | 555 |

**Cochrane Library**

| #1 | MeSH descriptor: [Kidney Failure, Chronic] explode all trees | 4249 |
| --- | --- | --- |
| #2 | MeSH descriptor: [Renal Replacement Therapy] explode all trees | 8258 |
| #3 | (((end stage or chronic) and (kidney diseas* or kidney failure* or renal disease* or renal failure*))):ti,ab,kw (Word variations have been searched) | 13772 |
| #4 | (((renal or kidney) and replace*)):ti,ab,kw (Word variations have been searched) | 2562 |
| #5 | ((hemodiafilt* or haemodiafilt* or hemodialys* or haemodialys*)):ti,ab,kw (Word variations have been searched) | 8577 |
| #6 | (dialys*):ti,ab,kw (Word variations have been searched) | 10560 |
| #7 | #1 OR #2 OR #3 OR #4 OR #5 OR #6 | 25359 |
| #8 | MeSH descriptor: [Conservative Treatment] this term only | 64 |
| #9 | MeSH descriptor: [Palliative Care] this term only | 1462 |
| #10 | MeSH descriptor: [Delivery of Health Care, Integrated] explode all trees | 323 |
| #11 | (((conservative or supportive or palliative) and (treatment* or management or care))):ti,ab,kw (Word variations have been searched) | 74043 |
| #12 | (integrated care):ti,ab,kw (Word variations have been searched) | 7018 |
| #13 | ((nondialys* or non-dialys*)):ti,ab,kw (Word variations have been searched) | 270 |
| #14 | #8 OR #9 OR #10 OR #11 OR #12 OR #13 | 78956 |
| #15 | MeSH descriptor: [Outcome and Process Assessment (Health Care)] explode all trees | 129827 |
| #16 | MeSH descriptor: [Prognosis] explode all trees | 138740 |
| #17 | MeSH descriptor: [Quality of Life] this term only | 20557 |
| #18 | MeSH descriptor: [Mortality] explode all trees | 12336 |
| #19 | MeSH descriptor: [Patient Readmission] this term only | 885 |
| #20 | MeSH descriptor: [Emotions] explode all trees | 15584 |
| #21 | MeSH descriptor: [Depression] this term only | 9685 |
| #22 | MeSH descriptor: [Clinical Decision-Making] this term only | 133 |
| #23 | MeSH descriptor: [Decision Making] explode all trees | 3732 |
| #24 | MeSH descriptor: [Life Expectancy] this term only | 121 |
| #25 | ((“quality of life” or qol or mortality or anxiety or depression)):ti,ab,kw (Word variations have been searched) | 197229 |
| #26 | ((decision* and (regret* or conflict*))):ti,ab,kw (Word variations have been searched) | 845 |
| #27 | (place of death):ti,ab,kw (Word variations have been searched) | 1273 |
| #28 | (symptom burden*):ti,ab,kw (Word variations have been searched) | 3303 |
| #29 | (life expectancy):ti,ab,kw (Word variations have been searched) | 7093 |
| #30 | #15 OR #16 OR #17 OR #18 OR #19 OR #20 OR #21 OR #22 OR #23 OR #24 OR #25 OR #26 OR #27 OR #28 OR #29 | 312943 |
| #31 | #7 AND #14 AND #30 | 888 |
| - | Cochrane reviews | 49 |

**CINAHL**

| # | Query | Limiters/Expanders | Results |
| --- | --- | --- | --- |
| S36 | S35 | Limiters - Language: Danish, English, Norwegian, Swedish  Search modes - Boolean/Phrase | 57 |
| S35 | S30 AND S34 | Search modes - Boolean/Phrase | 60 |
| S34 | S31 OR S32 OR S33 | Search modes - Boolean/Phrase | 279078 |
| S33 | pooled N1 (data or analys*) | Search modes - Boolean/Phrase | 6753 |
| S32 | ((systematic or method*) N3 (review* or overview* or study or studies or search* or approach*)) or meta analy* or meta-analy* or metaanaly* | Search modes - Boolean/Phrase | 268322 |
| S31 | PT (Systematic Review or Meta-Analysis) | Search modes - Boolean/Phrase | 87354 |
| S30 | S7 AND S13 AND S29 | Search modes - Boolean/Phrase | 640 |
| S29 | S14 OR S15 OR S16 OR S17 OR S18 OR S19 OR S20 OR S21 OR S22 OR S23 OR S24 OR S25 OR S26 OR S27 OR S28 | Search modes - Boolean/Phrase | 978656 |
| S28 | life expectancy | Search modes - Boolean/Phrase | 11238 |
| S27 | (MH “Life Expectancy”) | Search modes - Boolean/Phrase | 6408 |
| S26 | (symptom* N2 burden*) | Search modes - Boolean/Phrase | 2076 |
| S25 | place of death | Search modes - Boolean/Phrase | 594 |
| S24 | (decision* N2 (regret* or conflict*)) | Search modes - Boolean/Phrase | 935 |
| S23 | (“quality of life” or qol or mortality or anxiety or depression) | Search modes - Boolean/Phrase | 516601 |
| S22 | (MH “Decision Making+”) | Search modes - Boolean/Phrase | 100481 |
| S21 | (MH “Depression+”) | Search modes - Boolean/Phrase | 92524 |
| S20 | (MH “Emotions+”) | Search modes - Boolean/Phrase | 105698 |
| S19 | (MH “Readmission”) | Search modes - Boolean/Phrase | 10255 |
| S18 | (MH “Mortality+”) | Search modes - Boolean/Phrase | 57902 |
| S17 | (MH “Quality-Adjusted Life Years”) | Search modes - Boolean/Phrase | 3652 |
| S16 | (MH “Quality of Life+”) | Search modes - Boolean/Phrase | 95651 |
| S15 | (MH “Prognosis+”) | Search modes - Boolean/Phrase | 358121 |
| S14 | (MH “Process Assessment (Health Care)+”) OR (MH “Outcome Assessment”) | Search modes - Boolean/Phrase | 43482 |
| S13 | S8 OR S9 OR S10 OR S11 OR S12 | Search modes - Boolean/Phrase | 62938 |
| S12 | (nondialys* or non-dialys*) | Search modes - Boolean/Phrase | 409 |
| S11 | integrated care | Search modes - Boolean/Phrase | 3117 |
| S10 | ((conservative or supportive or palliative) N3 (treatment* or management or care)) | Search modes - Boolean/Phrase | 51873 |
| S9 | (MH “Health Care Delivery, Integrated”) | Search modes - Boolean/Phrase | 8919 |
| S8 | (MH “Palliative Care”) | Search modes - Boolean/Phrase | 30087 |
| S7 | S1 OR S2 OR S3 OR S4 OR S5 OR S6 | Search modes - Boolean/Phrase | 56906 |
| S6 | dialys* | Search modes - Boolean/Phrase | 21681 |
| S5 | (hemodiafilt* or haemodiafilt* or hemodialys* or haemodialys*) | Search modes - Boolean/Phrase | 18010 |
| S4 | ((renal or kidney) N3 replace*) | Search modes - Boolean/Phrase | 3989 |
| S3 | ((end stage or chronic) N3 (kidney diseas* or kidney failure* or renal disease* or renal failure*)) | Search modes - Boolean/Phrase | 30907 |
| S2 | (MH “Renal Replacement Therapy+”) | Search modes - Boolean/Phrase | 26424 |
| S1 | (MH “Kidney Failure, Chronic+”) | Search modes - Boolean/Phrase | 18573 |
