# Supplementary material for: Does conservative kidney management offer a quantity or quality of life benefit compared to dialysis? A systematic review
Source: BMC Nephrol. 2021 Sep 11;22:307. doi: 10.1186/s12882-021-02516-6 (PMC8434727; doi:10.1186/s12882-021-02516-6)

## Additional file 2: Flow Charts for Inclusion and Exclusion of Literature

# Flow chart for guidelines


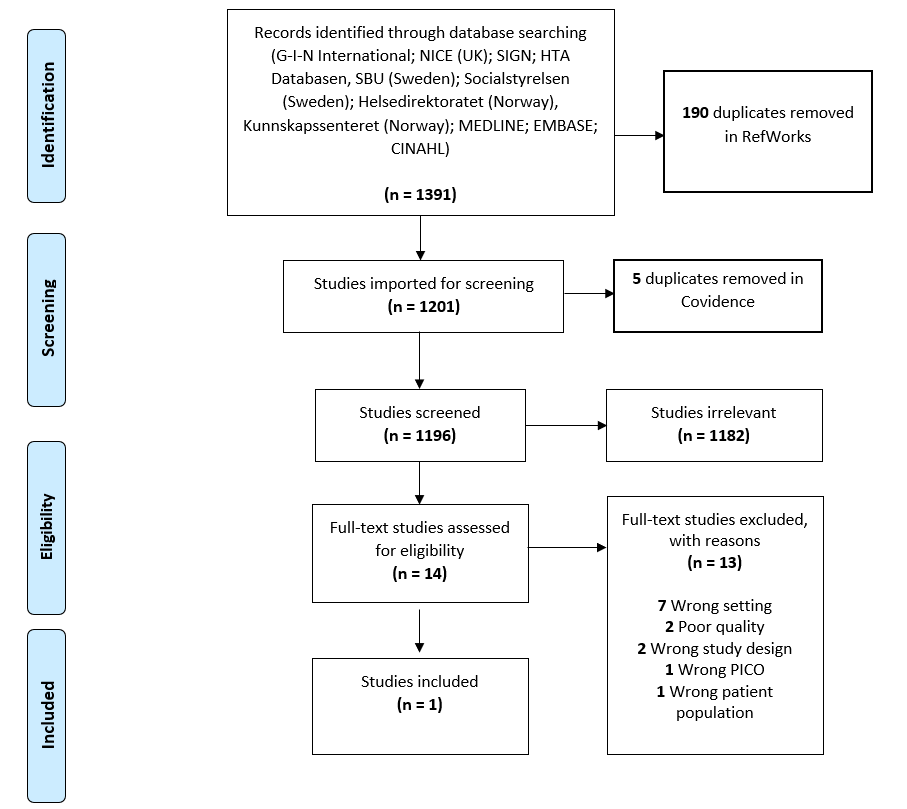


**Flow chart for reviews**


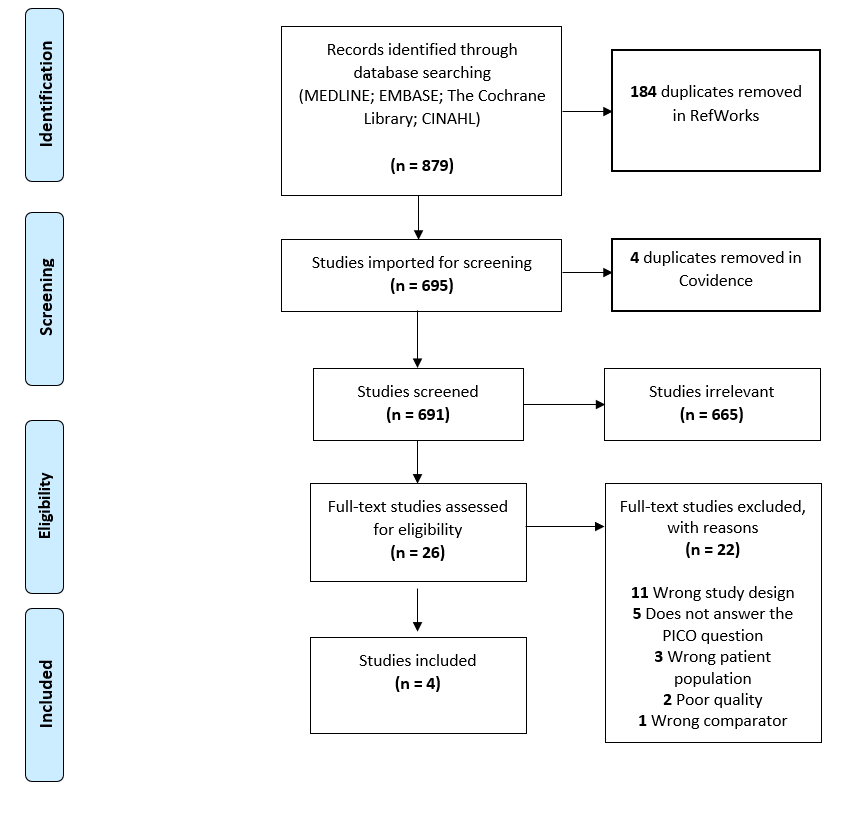

Supplement: Supplementary file 2 — Additional file 2: [file 12882_2021_2516_MOESM2_ESM.docx]
