# Supplementary material for: Does conservative kidney management offer a quantity or quality of life benefit compared to dialysis? A systematic review
Source: BMC Nephrol. 2021 Sep 11;22:307. doi: 10.1186/s12882-021-02516-6 (PMC8434727; doi:10.1186/s12882-021-02516-6)
Supplement: Supplementary file 3 — Additional file 3: [file 12882_2021_2516_MOESM3_ESM.docx]

Additional file 3: Overview of Primary Studies Included in Our Review Compared to Primary Studies Included in the European Guideline by Farrington and in Four Systematic Reviews

| Studies | Our review | Farrington 2016 (1) | Foote 2016 (2) | O'Connor 2012 (3) | Tsai 2017 (4) | Wongrakpanich 2017 (5) |
| --- | --- | --- | --- | --- | --- | --- |
| **Almutary 2016 (6)** | X |  |  |  |  |  |
| **Brown 2015 (7)** | X | X |  |  |  | X |
| **Carson 2009 (8)** | X | X | X | X |  | X |
| **Chan 2007 (9)** |  | X |  |  |  |  |
| **Chandna 2011 (10)** | X | X | X | X |  | X |
| **Da Silva-Gane 2012 (11)** | X | X | X |  | X | X |
| **De Biase 2008 (12)** |  | X |  | X | X | X |
| **Echevers 2016 (13)** |  | X |  |  |  |  |
| **Ellam 2009 (14)** |  | X | X | X |  |  |
| **G**rà**cia-Garcia 2012 (15)** |  | X |  |  |  |  |
| **Hussain 2013 (16)** | X | X | X |  |  | X |
| **Isaacs 2012 (17)** |  |  | X |  |  |  |
| **Iyasere 2019 (18)** | X |  |  |  |  |  |
| **Joly 2003 (19)** | X | X | X | X |  | X |
| **Kwok 2016 (20)** | X |  |  |  |  |  |
| **Murphy 2009 (21)** |  |  |  | X |  |  |
| **Murtagh 2007 (22)** | X | X | X | X |  | X |
| **Murtagh 2007 (23)** |  |  |  | X |  |  |
| **Murtagh 2010 (24)** |  | X |  | X |  |  |
| **Raman 2018 (25)** | X |  |  |  |  |  |
| **Reindl-Schwaighofer 2017 (26)** | X |  |  |  |  |  |
| **Rodriguez 2014 (27)** |  | X |  |  |  |  |
| **Saini 2006 (28)** |  |  |  | X |  |  |
| **Seow 2013 (29)** | X | X | X |  | X |  |
| **Shah 2019 (30)** | X |  |  |  |  |  |
| **Shih 2014 (31)** |  |  |  |  |  | X |
| **Shum 2014 (32)** | X | X | X |  |  | X |
| **Smith 2003 (33)** | X | X | X | X |  | X |
| **Tam-Tham 2018 (34)** | X |  |  |  |  |  |
| **Teo 2010 (35)** | X | X |  |  |  |  |
| **Teruel 2015 (36)** | X |  |  |  |  |  |
| **van Loon 2019 (37)** | X |  |  |  |  |  |
| **Verberne 2018 (38)** | X |  |  |  |  |  |
| **Verberne 2016 (39)** | X | X |  |  |  | X |
| **Wong 2007 (40)** |  | X | X | X |  |  |
| **Yong 2009 (41)** | X |  |  | X | X |  |

1. Farrington K, Covic A, Nistor I, Aucella F, Clyne N, De Vos L, et al. Clinical Practice Guideline on management of older patients with chronic kidney disease stage 3b or higher (eGFR<45 mL/min/1.73 m2): a summary document from the European Renal Best Practice Group. Nephrology, dialysis, transplantation : official publication of the European Dialysis and Transplant Association - European Renal Association. 2017;32(1):9-16.

2. Foote C, Kotwal S, Gallagher M, Cass A, Brown M, Jardine M. Survival outcomes of supportive care versus dialysis therapies for elderly patients with end-stage kidney disease: A systematic review and meta-analysis. Nephrology (Carlton, Vic). 2016;21(3):241-53.

3. O'Connor NR, Kumar P. Conservative management of end-stage renal disease without dialysis: a systematic review. Journal of palliative medicine. 2012;15(2):228-35.

4. Tsai HB, Chao CT, Chang RE, Hung KY, Group CS. Conservative management and health-related quality of life in end-stage renal disease: a systematic review. Clinical and investigative medicine Medecine clinique et experimentale. 2017;40(3):E127-e34.

5. Wongrakpanich S, Susantitaphong P, Isaranuwatchai S, Chenbhanich J, Eiam-Ong S, Jaber BL. Dialysis Therapy and Conservative Management of Advanced Chronic Kidney Disease in the Elderly: A Systematic Review. Nephron. 2017;137(3):178-89.

6. Almutary H, Bonner A, Douglas C. Which patients with chronic kidney disease have the greatest symptom burden? A comparative study of advanced CKD stage and dialysis modality. Journal of renal care. 2016;42(2):73-82.

7. Brown MA, Collett GK, Josland EA, Foote C, Li Q, Brennan FP. CKD in elderly patients managed without dialysis: survival, symptoms, and quality of life. Clinical journal of the American Society of Nephrology : CJASN. 2015;10(2):260-8.

8. Carson RC, Juszczak M, Davenport A, Burns A. Is maximum conservative management an equivalent treatment option to dialysis for elderly patients with significant comorbid disease? Clinical journal of the American Society of Nephrology : CJASN. 2009;4(10):1611-9.

9. Chan CH, Noble H, Lo SH, Kwan TH, Lee SL, Sze WK. Palliative care for patients with end-stage renal disease: experiences from Hong Kong. International journal of palliative nursing. 2007;13(7):310-4.

10. Chandna SM, Da Silva-Gane M, Marshall C, Warwicker P, Greenwood RN, Farrington K. Survival of elderly patients with stage 5 CKD: comparison of conservative management and renal replacement therapy. Nephrology, dialysis, transplantation : official publication of the European Dialysis and Transplant Association - European Renal Association. 2011;26(5):1608-14.

11. Da Silva-Gane M, Wellsted D, Greenshields H, Norton S, Chandna SM, Farrington K. Quality of life and survival in patients with advanced kidney failure managed conservatively or by dialysis. Clinical journal of the American Society of Nephrology : CJASN. 2012;7(12):2002-9.

12. De Biase V, Tobaldini O, Boaretti C, Abaterusso C, Pertica N, Loschiavo C, et al. Prolonged conservative treatment for frail elderly patients with end-stage renal disease: the Verona experience. Nephrology, dialysis, transplantation : official publication of the European Dialysis and Transplant Association - European Renal Association. 2008;23(4):1313-7.

13. Martínez Echevers Y, Toapanta Gaibor NG, Nava Pérez N, Barbosa Martin F, Montes Delgado R, Guerrero Riscos M. Survival of patients ≥70 years with advanced chronic kidney disease: Dialysis vs. conservative care. Nefrologia : publicacion oficial de la Sociedad Espanola Nefrologia. 2016;36(3):283-91.

14. Ellam T, El-Kossi M, Prasanth KC, El-Nahas M, Khwaja A. Conservatively managed patients with stage 5 chronic kidney disease--outcomes from a single center experience. QJM : monthly journal of the Association of Physicians. 2009;102(8):547-54.

15. Gràcia-Garcia S, Montañés-Bermúdez R, Morales-García LJ, Díez-de Los Ríos MJ, Jiménez-García J, Macías-Blanco C, et al. Current use of equations for estimating glomerular filtration rate in Spanish laboratories. Nefrologia : publicacion oficial de la Sociedad Espanola Nefrologia. 2012;32(4):508-16.

16. Hussain JA, Mooney A, Russon L. Comparison of survival analysis and palliative care involvement in patients aged over 70 years choosing conservative management or renal replacement therapy in advanced chronic kidney disease. Palliative medicine. 2013;27(9):829-39.

17. Isaacs A, Burns A, Davenport A. Is dialysis a viable option for the older patient? Outcomes for patients starting dialysis aged 80 years or older. Blood purification. 2012;33(4):257-62.

18. Iyasere O, Brown EA, Johansson L, Davenport A, Farrington K, Maxwell AP, et al. Quality of life with conservative care compared with assisted peritoneal dialysis and haemodialysis. Clinical kidney journal. 2019;12(2):262-8.

19. Joly D, Anglicheau D, Alberti C, Nguyen AT, Touam M, Grunfeld JP, et al. Octogenarians reaching end-stage renal disease: cohort study of decision-making and clinical outcomes. J Am Soc Nephrol. 2003;14(4):1012-21.

20. Kwok WH, Yong SP, Kwok OL. Outcomes in elderly patients with end-stage renal disease: Comparison of renal replacement therapy and conservative management. Hong Kong Journal of Nephrology. 2016;19:42-56.

21. Murphy EL, Murtagh FE, Carey I, Sheerin NS. Understanding symptoms in patients with advanced chronic kidney disease managed without dialysis: use of a short patient-completed assessment tool. Nephron Clinical practice. 2009;111(1):c74-80.

22. Murtagh FE, Marsh JE, Donohoe P, Ekbal NJ, Sheerin NS, Harris FE. Dialysis or not? A comparative survival study of patients over 75 years with chronic kidney disease stage 5. Nephrology, dialysis, transplantation : official publication of the European Dialysis and Transplant Association - European Renal Association. 2007;22(7):1955-62.

23. Murtagh FE, Addington-Hall JM, Edmonds PM, Donohoe P, Carey I, Jenkins K, et al. Symptoms in advanced renal disease: a cross-sectional survey of symptom prevalence in stage 5 chronic kidney disease managed without dialysis. Journal of palliative medicine. 2007;10(6):1266-76.

24. Murtagh FE, Addington-Hall J, Edmonds P, Donohoe P, Carey I, Jenkins K, et al. Symptoms in the month before death for stage 5 chronic kidney disease patients managed without dialysis. Journal of pain and symptom management. 2010;40(3):342-52.

25. Raman M, Middleton RJ, Kalra PA, Green D. Outcomes in dialysis versus conservative care for older patients: A prospective cohort analysis of stage 5 Chronic Kidney Disease. PLoS One. 2018;13(10):e0206469.

26. Reindl-Schwaighofer R, Kainz A, Kammer M, Dumfarth A, Oberbauer R. Survival analysis of conservative vs. dialysis treatment of elderly patients with CKD stage 5. PLoS One. 2017;12(7):e0181345.

27. Rodriguez Villarreal I, Ortega O, Hinostroza J, Cobo G, Gallar P, Mon C, et al. Geriatric assessment for therapeutic decision-making regarding renal replacement in elderly patients with advanced chronic kidney disease. Nephron Clinical practice. 2014;128(1-2):73-8.

28. Saini T, Murtagh FE, Dupont PJ, McKinnon PM, Hatfield P, Saunders Y. Comparative pilot study of symptoms and quality of life in cancer patients and patients with end stage renal disease. Palliative medicine. 2006;20(6):631-6.

29. Seow YY, Cheung YB, Qu LM, Yee AC. Trajectory of quality of life for poor prognosis stage 5D chronic kidney disease with and without dialysis. American journal of nephrology. 2013;37(3):231-8.

30. Shah KK, Murtagh FEM, McGeechan K, Crail S, Burns A, Tran AD, et al. Health-related quality of life and well-being in people over 75 years of age with end-stage kidney disease managed with dialysis or comprehensive conservative care: a cross-sectional study in the UK and Australia. BMJ open. 2019;9(5):e027776.

31. Shih CJ, Chen YT, Ou SM, Yang WC, Kuo SC, Tarng DC. The impact of dialysis therapy on older patients with advanced chronic kidney disease: a nationwide population-based study. BMC medicine. 2014;12:169.

32. Shum CK, Tam KF, Chak WL, Chan TC, Mak YF, Chau KF. Outcomes in older adults with stage 5 chronic kidney disease: comparison of peritoneal dialysis and conservative management. The journals of gerontology Series A, Biological sciences and medical sciences. 2014;69(3):308-14.

33. Smith C, Da Silva-Gane M, Chandna S, Warwicker P, Greenwood R, Farrington K. Choosing not to dialyse: evaluation of planned non-dialytic management in a cohort of patients with end-stage renal failure. Nephron Clinical practice. 2003;95(2):c40-6.

34. Tam-Tham H, Quinn RR, Weaver RG, Zhang J, Ravani P, Liu P, et al. Survival among older adults with kidney failure is better in the first three years with chronic dialysis treatment than not. Kidney international. 2018;94(3):582-8.

35. Teo BW, Ma V, Xu H, Li J, Lee EJ. Profile of hospitalisation and death in the first year after diagnosis of end-stage renal disease in a multi-ethnic Asian population. Annals of the Academy of Medicine, Singapore. 2010;39(2):79-87.

36. Teruel JL, Burguera Vion V, Gomis Couto A, Rivera Gorrin M, Fernandez-Lucas M, Rodriguez Mendiola N, et al. Choosing conservative therapy in chronic kidney disease. Nefrologia : publicacion oficial de la Sociedad Espanola Nefrologia. 2015;35(3):273-9.

37. van Loon IN, Goto NA, Boereboom FTJ, Verhaar MC, Bots ML, Hamaker ME. Quality of life after the initiation of dialysis or maximal conservative management in elderly patients: a longitudinal analysis of the Geriatric assessment in OLder patients starting Dialysis (GOLD) study. BMC nephrology. 2019;20(1):108.

38. Verberne WR, Dijkers J, Kelder JC, Geers ABM, Jellema WT, Vincent HH, et al. Value-based evaluation of dialysis versus conservative care in older patients with advanced chronic kidney disease: a cohort study. BMC nephrology. 2018;19(1):205.

39. Verberne WR, Geers AB, Jellema WT, Vincent HH, van Delden JJ, Bos WJ. Comparative Survival among Older Adults with Advanced Kidney Disease Managed Conservatively Versus with Dialysis. Clinical journal of the American Society of Nephrology : CJASN. 2016;11(4):633-40.

40. Wong CF, McCarthy M, Howse ML, Williams PS. Factors affecting survival in advanced chronic kidney disease patients who choose not to receive dialysis. Renal failure. 2007;29(6):653-9.

41. Yong DS, Kwok AO, Wong DM, Suen MH, Chen WT, Tse DM. Symptom burden and quality of life in end-stage renal disease: a study of 179 patients on dialysis and palliative care. Palliative medicine. 2009;23(2):111-9.
