# Supplementary material for: Does conservative kidney management offer a quantity or quality of life benefit compared to dialysis? A systematic review
Source: BMC Nephrol. 2021 Sep 11;22:307. doi: 10.1186/s12882-021-02516-6 (PMC8434727; doi:10.1186/s12882-021-02516-6)
Supplement: Supplementary file 4 — Additional file 4: [file 12882_2021_2516_MOESM4_ESM.docx]

Additional file 4: Reasons Why Primary Studies Included in the European guideline by Farrington and in Four Systematic Reviews are Not Included in Our Review

| Studies | Reason for exclusion |
| --- | --- |
| **Chan 2007 (1)** | No comparator: only CKM patients |
| **De Biase 2008 (2)** | Low quality of evidence: does not report p value, SD or CI |
| **Echevers 2016 (3)** | Different language: only in Spanish |
| **Ellam 2009 (4)** | No comparator: only CKM patients |
| **Gràcia-Garcia 2012 (5)** | No comparator: only CKM patients |
| **Isaacs 2012 (6)** | No comparator: only dialysis patients, Polish register report |
| **Murphy 2009 (7)** | No comparator: only CKM patients |
| **Murtagh 2007 (8)** | No comparator: only CKM patients |
| **Murtagh 2010 (9)** | No comparator: only CKM patients |
| **Rodriguez 2014 (10)** | Different outcome: which patients opt for CKM and which patients opt for dialysis |
| **Saini 2006 (11)** | Different comparator: comparing CKM patients with terminally ill malignancy patients |
| **Shih 2014 (12)** | Different definition of CKM patients: Include all patients above 70 years with creatinine levels >6 mg/dl who had been treated with erythropoiesis-stimulating agents |
| **Wong 2007 (13)** | No comparator: only CKM patients |

1. Chan CH, Noble H, Lo SH, Kwan TH, Lee SL, Sze WK. Palliative care for patients with end-stage renal disease: experiences from Hong Kong. International journal of palliative nursing. 2007;13(7):310-4.

2. De Biase V, Tobaldini O, Boaretti C, Abaterusso C, Pertica N, Loschiavo C, et al. Prolonged conservative treatment for frail elderly patients with end-stage renal disease: the Verona experience. Nephrology, dialysis, transplantation : official publication of the European Dialysis and Transplant Association - European Renal Association. 2008;23(4):1313-7.

3. Martínez Echevers Y, Toapanta Gaibor NG, Nava Pérez N, Barbosa Martin F, Montes Delgado R, Guerrero Riscos M. Survival of patients ≥70 years with advanced chronic kidney disease: Dialysis vs. conservative care. Nefrologia : publicacion oficial de la Sociedad Espanola Nefrologia. 2016;36(3):283-91.

4. Ellam T, El-Kossi M, Prasanth KC, El-Nahas M, Khwaja A. Conservatively managed patients with stage 5 chronic kidney disease--outcomes from a single center experience. QJM : monthly journal of the Association of Physicians. 2009;102(8):547-54.

5. Gràcia-Garcia S, Montañés-Bermúdez R, Morales-García LJ, Díez-de Los Ríos MJ, Jiménez-García J, Macías-Blanco C, et al. Current use of equations for estimating glomerular filtration rate in Spanish laboratories. Nefrologia : publicacion oficial de la Sociedad Espanola Nefrologia. 2012;32(4):508-16.

6. Isaacs A, Burns A, Davenport A. Is dialysis a viable option for the older patient? Outcomes for patients starting dialysis aged 80 years or older. Blood purification. 2012;33(4):257-62.

7. Murphy EL, Murtagh FE, Carey I, Sheerin NS. Understanding symptoms in patients with advanced chronic kidney disease managed without dialysis: use of a short patient-completed assessment tool. Nephron Clinical practice. 2009;111(1):c74-80.

8. Murtagh FE, Addington-Hall JM, Edmonds PM, Donohoe P, Carey I, Jenkins K, et al. Symptoms in advanced renal disease: a cross-sectional survey of symptom prevalence in stage 5 chronic kidney disease managed without dialysis. Journal of palliative medicine. 2007;10(6):1266-76.

9. Murtagh FE, Addington-Hall J, Edmonds P, Donohoe P, Carey I, Jenkins K, et al. Symptoms in the month before death for stage 5 chronic kidney disease patients managed without dialysis. Journal of pain and symptom management. 2010;40(3):342-52.

10. Rodriguez Villarreal I, Ortega O, Hinostroza J, Cobo G, Gallar P, Mon C, et al. Geriatric assessment for therapeutic decision-making regarding renal replacement in elderly patients with advanced chronic kidney disease. Nephron Clinical practice. 2014;128(1-2):73-8.

11. Saini T, Murtagh FE, Dupont PJ, McKinnon PM, Hatfield P, Saunders Y. Comparative pilot study of symptoms and quality of life in cancer patients and patients with end stage renal disease. Palliative medicine. 2006;20(6):631-6.

12. Shih CJ, Chen YT, Ou SM, Yang WC, Kuo SC, Tarng DC. The impact of dialysis therapy on older patients with advanced chronic kidney disease: a nationwide population-based study. BMC medicine. 2014;12:169.

13. Wong CF, McCarthy M, Howse ML, Williams PS. Factors affecting survival in advanced chronic kidney disease patients who choose not to receive dialysis. Renal failure. 2007;29(6):653-9.
