# Supplementary material for: Does conservative kidney management offer a quantity or quality of life benefit compared to dialysis? A systematic review
Source: BMC Nephrol. 2021 Sep 11;22:307. doi: 10.1186/s12882-021-02516-6 (PMC8434727; doi:10.1186/s12882-021-02516-6)
Supplement: Supplementary file 5 — Additional file 5: [file 12882_2021_2516_MOESM5_ESM.docx]

Additional file 5: Quality Assessment of Primary Studies by ROBINS-I Tool – Consensus between Reviewers

| **Study** | **Risk of bias due to confounding** | **Risk of bias in selection of participants for the study** | **Risk of bias in classification of interventions** | \| **Risk of bias due to deviations from intended interventions** \| \| --- \| | **Risk of bias due to missing data** | \| **Risk of bias in measurement of outcomes** \|  \| \| --- \| --- \| | **Risk of bias in selection of the reported result** | **Overall risk of bias** | **Direction of bias for this outcome** |
| --- | --- | --- | --- | --- | --- | --- | --- | --- | --- | --- | --- | --- |
| **Almutary 2016 (1)** | Critical | Critical | Low | Low | Low | Low | Low | Serious | Unpredictable |
| **Brown 2015 (2)** | Moderate | Low | Low | Low | Moderate | Low | Low | Moderate | Favours comparator |
| **Carson 2009 (3)** | Serious | Serious | Low | Low | Low | Moderate | Low | Serious | Unpredictable |
| **Chandna 2011 (4)** | Low | Low | Low | Low | Low | Moderate | Low | Moderate | Favours comparator |
| **Da Silva-Gane 2012 (5)** | Low | Low | Low | Low | Low | Moderate | Low | Moderate | Favours comparator |
| **Hussain 2013 (6)** | Critical | Moderate | Low | Low | Moderate | Moderate | Low | Serious | Favours comparator |
| **Iyasere 2019 (7)** | Moderate | Low | Low | Low | Low | Low | Low | Moderate | Unpredictable |
| **Joly 2003 (8)** | Low | Low | Low | Low | Low | Low | Low | Low | Towards null |
| **Kwok 2016 (9)** | Serious | Low | Low | Low | Low | Low | Low | Moderate | Favours comparator |
| **Murtagh 2007 (10)** | Low | Moderate | Moderate | Low | Low | Moderate | Low | Serious | Favours comparator |
| **Raman 2018 (11)** | Low | Low | Low | Low | Low | Moderate | Low | Moderate | Unpredictable |
| **Reindl-Schwaighofer 2017 (12)** | Low | Moderate | Low | Low | Low | Moderate | Low | Moderate | Unpredictable |
| **Seow 2013 (13)** | Serious | Serious | Low | Moderate | Moderate | Moderate | Low | Serious | Favours intervention |
| **Shah 2019 (14)** | Critical | Low | Low | Low | Moderate | Moderate | Low | Serious | Unpredictable |
| **Shum 2014 (15)** | Moderate | Moderate | Low | Low | Low | Moderate | Low | Serious | Favours comparator |
| **Smith 2003 (16)** | Moderate | Low | Low | Low | Low | Moderate | Low | Moderate | Favours comparator |
| **Tam-Tham 2018 (17)** | Moderate | Moderate | Low | Low | Low | Moderate | Low | Serious | Towards null |
| **Tan 2017 (18)** | Critical | Low | Low | Low | Low | Moderate | Moderate | Critical | Unpredictable |
| **Teo 2010 (19)** | Low | Low | Low | Low | Low | Moderate | Serious | Moderate | Unpredictable |
| **Teruel 2015 (20)** | Moderate | Moderate | Low | Low | Low | Moderate | Low | Moderate | Unpredictable |
| **van Loon**  **2019 (21)** | Low | Low | Low | Moderate | Low | Low | Moderate | Low | Towards null |
| **Verberne 2016 (22)** | Low | Moderate | Low | Low | Low | Moderate | Low | Moderate | Unpredictable |
| **Verberne 2018 (23)** | Low | Moderate | Low | Low | Low | Moderate | Low | Moderate | Unpredictable |
| **Yong 2009 (24)** | Critical | Low | Low | Low | Low | Moderate | Low | Serious | Favours comparator |
| **Yuen 2016 (25)** | Low | Low | Low | Low | Low | Low | Low | Low | Favours comparator |

1. Almutary H, Bonner A, Douglas C. Which patients with chronic kidney disease have the greatest symptom burden? A comparative study of advanced CKD stage and dialysis modality. Journal of renal care. 2016;42(2):73-82.

2. Brown MA, Collett GK, Josland EA, Foote C, Li Q, Brennan FP. CKD in elderly patients managed without dialysis: survival, symptoms, and quality of life. Clinical journal of the American Society of Nephrology : CJASN. 2015;10(2):260-8.

3. Carson RC, Juszczak M, Davenport A, Burns A. Is maximum conservative management an equivalent treatment option to dialysis for elderly patients with significant comorbid disease? Clinical journal of the American Society of Nephrology : CJASN. 2009;4(10):1611-9.

4. Chandna SM, Da Silva-Gane M, Marshall C, Warwicker P, Greenwood RN, Farrington K. Survival of elderly patients with stage 5 CKD: comparison of conservative management and renal replacement therapy. Nephrology, dialysis, transplantation : official publication of the European Dialysis and Transplant Association - European Renal Association. 2011;26(5):1608-14.

5. Da Silva-Gane M, Wellsted D, Greenshields H, Norton S, Chandna SM, Farrington K. Quality of life and survival in patients with advanced kidney failure managed conservatively or by dialysis. Clinical journal of the American Society of Nephrology : CJASN. 2012;7(12):2002-9.

6. Hussain JA, Mooney A, Russon L. Comparison of survival analysis and palliative care involvement in patients aged over 70 years choosing conservative management or renal replacement therapy in advanced chronic kidney disease. Palliative medicine. 2013;27(9):829-39.

7. Iyasere O, Brown EA, Johansson L, Davenport A, Farrington K, Maxwell AP, et al. Quality of life with conservative care compared with assisted peritoneal dialysis and haemodialysis. Clinical kidney journal. 2019;12(2):262-8.

8. Joly D, Anglicheau D, Alberti C, Nguyen AT, Touam M, Grunfeld JP, et al. Octogenarians reaching end-stage renal disease: cohort study of decision-making and clinical outcomes. J Am Soc Nephrol. 2003;14(4):1012-21.

9. Kwok WH, Yong SP, Kwok OL. Outcomes in elderly patients with end-stage renal disease: Comparison of renal replacement therapy and conservative management. Hong Kong Journal of Nephrology. 2016;19:42-56.

10. Murtagh FE, Marsh JE, Donohoe P, Ekbal NJ, Sheerin NS, Harris FE. Dialysis or not? A comparative survival study of patients over 75 years with chronic kidney disease stage 5. Nephrology, dialysis, transplantation : official publication of the European Dialysis and Transplant Association - European Renal Association. 2007;22(7):1955-62.

11. Raman M, Middleton RJ, Kalra PA, Green D. Outcomes in dialysis versus conservative care for older patients: A prospective cohort analysis of stage 5 Chronic Kidney Disease. PLoS One. 2018;13(10):e0206469.

12. Reindl-Schwaighofer R, Kainz A, Kammer M, Dumfarth A, Oberbauer R. Survival analysis of conservative vs. dialysis treatment of elderly patients with CKD stage 5. PLoS One. 2017;12(7):e0181345.

13. Seow YY, Cheung YB, Qu LM, Yee AC. Trajectory of quality of life for poor prognosis stage 5D chronic kidney disease with and without dialysis. American journal of nephrology. 2013;37(3):231-8.

14. Shah KK, Murtagh FEM, McGeechan K, Crail S, Burns A, Tran AD, et al. Health-related quality of life and well-being in people over 75 years of age with end-stage kidney disease managed with dialysis or comprehensive conservative care: a cross-sectional study in the UK and Australia. BMJ open. 2019;9(5):e027776.

15. Shum CK, Tam KF, Chak WL, Chan TC, Mak YF, Chau KF. Outcomes in older adults with stage 5 chronic kidney disease: comparison of peritoneal dialysis and conservative management. The journals of gerontology Series A, Biological sciences and medical sciences. 2014;69(3):308-14.

16. Smith C, Da Silva-Gane M, Chandna S, Warwicker P, Greenwood R, Farrington K. Choosing not to dialyse: evaluation of planned non-dialytic management in a cohort of patients with end-stage renal failure. Nephron Clinical practice. 2003;95(2):c40-6.

17. Tam-Tham H, Quinn RR, Weaver RG, Zhang J, Ravani P, Liu P, et al. Survival among older adults with kidney failure is better in the first three years with chronic dialysis treatment than not. Kidney international. 2018;94(3):582-8.

18. Tan T, Brennan F, Brown MA. Impact of dialysis on symptom burden and functional state in the elderly. Renal Society of Australasia Journal. March, 2017;13(1):22-30.

19. Teo BW, Ma V, Xu H, Li J, Lee EJ. Profile of hospitalisation and death in the first year after diagnosis of end-stage renal disease in a multi-ethnic Asian population. Annals of the Academy of Medicine, Singapore. 2010;39(2):79-87.

20. Teruel JL, Burguera Vion V, Gomis Couto A, Rivera Gorrin M, Fernandez-Lucas M, Rodriguez Mendiola N, et al. Choosing conservative therapy in chronic kidney disease. Nefrologia : publicacion oficial de la Sociedad Espanola Nefrologia. 2015;35(3):273-9.

21. van Loon IN, Goto NA, Boereboom FTJ, Verhaar MC, Bots ML, Hamaker ME. Quality of life after the initiation of dialysis or maximal conservative management in elderly patients: a longitudinal analysis of the Geriatric assessment in OLder patients starting Dialysis (GOLD) study. BMC nephrology. 2019;20(1):108.

22. Verberne WR, Geers AB, Jellema WT, Vincent HH, van Delden JJ, Bos WJ. Comparative Survival among Older Adults with Advanced Kidney Disease Managed Conservatively Versus with Dialysis. Clinical journal of the American Society of Nephrology : CJASN. 2016;11(4):633-40.

23. Verberne WR, Dijkers J, Kelder JC, Geers ABM, Jellema WT, Vincent HH, et al. Value-based evaluation of dialysis versus conservative care in older patients with advanced chronic kidney disease: a cohort study. BMC nephrology. 2018;19(1):205.

24. Yong DS, Kwok AO, Wong DM, Suen MH, Chen WT, Tse DM. Symptom burden and quality of life in end-stage renal disease: a study of 179 patients on dialysis and palliative care. Palliative medicine. 2009;23(2):111-9.

25. Yuen S, Suen HP, Kwok O, Yong S, Tse M. Advance care planning for 600 Chinese patients with end-stage renal disease. Hong Kong Journal of Nephrology. 2016;19:19-27.
